# Fetal Alcohol Spectrum Disorder Prevalence Studies in School Settings: A Global Systematic Review of Methods, Measures, Strengths and Limitations

**DOI:** 10.1111/dar.70233

**Published:** 2026-09-10

**Authors:** Robyn McCarthy, Joshua Pink, Leana Olivier, David Gilbert, Raja Mukherjee, Penny A. Cook

**Affiliations:** ^1^ School of Health and Society University of Salford Salford UK; ^2^ Centre for Digital Innovations in Health & Social Care University of Bradford Bradford UK; ^3^ Foundation for Alcohol Related Research Cape Town South Africa; ^4^ Surrey and Borders Partnership NHS Foundation Trust Redhill UK

**Keywords:** active case ascertainment, alcohol, education, FASD, methodology

## Abstract

**Issues:**

Fetal alcohol spectrum disorder (FASD) is a prevalent yet widely underdiagnosed neurodevelopmental condition resulting from prenatal alcohol exposure (PAE). FASD has profound lifelong implications for affected individuals, their caregivers and society. Active case ascertainment is the gold standard for estimating FASD prevalence, but methodological variability limits comparability across studies. This review aims to systematically evaluate the methodologies and reporting practices of school‐based FASD prevalence studies, with the goal of identifying best practices and informing standardised approaches to enhance the validity, reproducibility and policy relevance of future research.

**Approach:**

A systematic review was conducted to examine the methods, measures, strengths and limitations of school‐based FASD prevalence studies. Five databases were searched using key terms associated with FASD prevalence in school settings. Identified studies were screened and data were extracted and analysed.

**Key Findings:**

The 29 included studies revealed variability in case definitions, diagnostic frameworks, tools and measures. Most studies employed active case ascertainment designs. Non‐participation rates and details on community engagement or co‐creation activities were often underreported. PAE assessment predominantly relied on maternal self‐report.

**Implications:**

Despite a shared goal of identifying FASD, methodological variability across studies may limit the comparability of outcomes. A lack of robust prevalence data impedes adequate funding for prevention, diagnosis and support services.

**Conclusion:**

Establishing a universally accepted case definition and diagnostic guidelines for FASD is essential. Reporting of community engagement and involvement of individuals with lived experience should become standard practice and the development of objective measures for PAE identification is increasingly necessary.

## Key Points

- Methodological inconsistencies limit comparability of prevalence figures.
- Need for a standardised yet locally and culturally appropriate diagnostic framework.
- Importance of reporting of community involvement and other inclusive research practices.

## 1 Introduction

Consumption of alcohol during pregnancy is a global issue with the most recent estimate being 9.8% (95% confidence interval 8.9–11.1) of pregnancies being alcohol exposed [1], while rates are as high as 40% in parts of Europe and 20% in parts of Africa [1]. While a decline in alcohol consumption has been observed in developed countries over the past 30 years [2], there has been an increase in emerging economies [3]. Prenatal alcohol exposure (PAE) remains a public health issue [4] with increasing evidence of risk from even low levels of alcohol consumption [5, 6]. PAE can lead to fetal alcohol spectrum disorder (FASD) and multiple worse health, educational and social outcomes for those affected, contributing to health inequity alongside other mechanisms of alcohol harm [7].

FASD is a lifelong neurodevelopmental condition that is caused when alcohol consumed during pregnancy crosses the placenta, affecting the development of the fetus [8, 9]. FASD is characterised by cognitive and behavioural impairments and, in some cases, alterations to facial features. Importantly, while these sentinel facial features are highly specific to FASD, many individuals with FASD do not present with sentinel facial features but nonetheless experience significant neurodevelopmental impairment.

FASD is an umbrella term used for a number of different diagnoses and diagnostic approaches which differ internationally. In Australia a dichotomous system classifies individuals as either FASD with sentinel facial features or without sentinel facial features [10], an approach also adopted in Canada [11], the UK via the Scottish SIGN guidelines [12] and in New Zealand [13]. In the United States Hoyme et al. [8] and the 4digit guidance [14] use a multi category framework instead, this distinguishes fetal alcohol syndrome (FAS), partial fetal alcohol syndrome, alcohol‐related neurodevelopmental disorder and alcohol‐related birth defects. In South Africa a gestalt approach is used for diagnosis recognising that the whole picture should be considered alongside the Hoyme et al. [8] diagnostic guidelines [15].

While these diagnostic systems differ in structure and terminology, they share recognition that sentinel facial features are not required for an FASD diagnosis and that significant neurodevelopmental impairment may occur without the presence of these facial features. However, variation in how this principle is operationalised across jurisdictions may contribute to inconsistency in identification and reporting in prevalence studies [16, 17].

Challenges in consistency of classification of FASD are compounded by underreporting of alcohol consumption during pregnancy due to stigma and social desirability bias [18], as well as the absence of routine recording of prenatal alcohol exposure in health records. Despite these limitations, prevalence estimates suggest that approximately 0.8% of the global population is affected by FASD, with higher rates reported in Europe [19]. Ongoing uncertainty around prevalence contributes to underinvestment in prevention and support services [17]. Active case ascertainment studies, the basis of prevalence estimates [19, 20], are considered the ‘gold standard’ method of estimating prevalence and involve screening a cross‐section of the general population [21]. Typically, active case ascertainment involves screening a large population using defined criteria or case definition, with individuals who meet predetermined thresholds invited for detailed multidisciplinary assessment to confirm or exclude the likely presence of FASD. This approach enables the identification of previously unrecognised cases and reduces reliance on referrals, thereby generating more accurate prevalence estimates [20]. Passive methods are less useful because individuals with FASD are often not diagnosed [22, 23] for a number of reasons, including a lack of awareness among relevant professionals [24] and difficulty in differentiating features of FASD from other commonly co‐occurring disorders (e.g., attention deficit hyperactivity disorder or autism) [25].

Children with FASD frequently experience learning difficulties, behavioural challenges and special educational needs and are therefore overrepresented in educational support services [26]. Teachers with knowledge of FASD have been found to better provide tailored support, reducing the risk of adverse educational outcomes [27]. School‐based active case ascertainment offers a unique opportunity to identify children who might otherwise remain undiagnosed, yet such studies raise important methodological, ethical and practical considerations, including consent processes, cultural sensitivity and engagement with families and communities.

There is growing recognition of the value of co‐creation, public involvement and community engagement in research, particularly when addressing culturally sensitive issues such as alcohol‐related harm [28]. Involving the relevant populations and communities in the design and implementation of research can make it more accurate and the findings more acceptable [29]. Co‐creation approaches may also help to mitigate stigma, build trust and reduce power imbalances between researchers and participants, particularly in marginalised or underserved populations. In the context of FASD research, co‐creation has been shown to support a more nuanced understanding of lived experience, improve recruitment and retention and facilitate more accurate interpretation of findings [30]. This review provides additional insight within FASD research by exploring methodologies that consider co‐creation in their methodology.

In school‐based FASD prevalence research, engagement with children, families, schools and communities may impact methodological considerations and may directly influence study feasibility, consent processes, recruitment, retention and acceptability [31]. This review sought to assess whether or not co‐creation, public involvement and community engagement were reported in studies that aimed to identify FASD in school settings as reporting provides the primary means by which the extent and transparency of these practices can be evaluated within published research [32].

Previous reviews have examined the prevalence of FASD, Roozen et al. [33] and Lange et al. [19] synthesised FASD prevalence studies in the general population. Subsequent reviews have applied Lange et al.'s estimates to a meta‐analysis of alcohol estimates [34] or have reviewed prevalence in special populations [35]. Examination of the studies included in Lange et al.'s [19] review indicates that the majority of general‐population prevalence estimates were derived from school‐based studies using active case ascertainment. Schools provide access to large, age‐defined cohorts and, as a result, school‐based active case ascertainment has become the primary approach for identifying FASD in population‐level research. Despite this central role, there has been no systematic synthesis of the methodological approaches used within school‐based studies, nor of the implications of methodological variability for prevalence estimation. Specifically, issues relating to study design, implementation challenges and the integration of co‐creation and public involvement have not been systematically examined. Focusing on school‐based studies allows for methodological comparability by holding the research setting constant, while also addressing a critical public health gap, as FASD is prevalent among students in mainstream schools yet remains substantially underdiagnosed within educational systems.

The present review synthesises the methodologies used in school‐based FASD identification studies, with particular attention to active case ascertainment approaches and the reporting of co‐creation and community engagement, in order to inform future research and provide evidence‐based recommendations to improve the identification of FASD within school contexts.

## 2 Methods

With a view to identifying all studies screening for FASD in a school setting, literature searches were conducted in five databases (CINAHL, MEDLINE, PsycINFO, Child Development & Adolescent Studies, Cochrane Library), with the searches initially conducted on 10 July 2023 and updated on 15 January 2026. Studies published from 2003 onwards were included, in an attempt to reflect the period during which FASD began to be more consistently conceptualised as a spectrum of neurodevelopmental impairment rather than solely as FAS [36]. Earlier studies often relied on older diagnostic frameworks focused on the classic FAS phenotype, limiting comparability with contemporary prevalence methodologies. The strategy contained three sets of terms: one set of terms for FASD, one set to capture studies conducted in school settings and one set to cover screening studies and related study types. Full details of the search terms and search strategy used are given in Appendix S1.

Search results obtained from each database were imported into the Covidence systematic review tool and automatically deduplicated. The titles and abstracts were then independently screened by two reviewers (R.Mc. and J.P.). Any discrepancies at this stage were resolved first by discussion between the reviewers and any outstanding disagreements were reconciled by a third reviewer (D.G.). Following this, all papers included after title and abstract screening had their full texts screened by the same two reviewers (R.Mc. and J.P.). As before, discrepancies were resolved first by discussion between the reviewers and any outstanding disagreements were reconciled by a third reviewer (D.G.). The reference lists of studies included at this stage were also checked for any additional references not identified through the original database search.

The final included studies were divided equally between two reviewers (R.Mc. and J.P.) who extracted data independently for their assigned studies using a pre‐specified data extraction template. The data extracted included: study design; country study conducted in; number of stages in screening design; whether height was used as a pre‐screening variable, whether weight used as a pre‐screening variable; whether head size was used as a pre‐screening variable; and whether parent opt‐in was used as a pre‐screening variables.

Studies were assigned randomly to the two authors, except for 1 paper on which R.Mc. is an author, which was therefore assigned to J.P. All extracted data were then cross‐checked by the other reviewer to ensure accuracy and consistency in data extraction.

The inclusion and exclusion criteria used are given in Table 1.

**TABLE 1:** Inclusion criteria for review.

Concept	Review criteria
Population	Inclusion: Children and adolescents (aged 5 to 18 years) attending school. Exclusion: Children under the age of 5, children not attending a school, adults.
Interventions	Screening strategies for FASD—Any structured tests, diagnostics or interventions used to identify undiagnosed cases of FASD in a school setting.
Comparators	Alternative screening strategies (if a study compares multiple different approaches to screening). No screening (if a study compares a screening intervention to no screening). Single arm studies were included if they meet the intervention criteria above.
Study designs	Randomised controlled trials Non‐randomised controlled trials Cohort studies Case‐control studies Cross‐sectional studies Before and after studies Prevalence studies Interrupted time series
Date of publication	Published between the years 2003–2026 and must be published in the English language

Abbreviation: FASD, fetal alcohol spectrum disorder.

The Quality Assessment of Diagnostic Accuracy Studies 2 (QUADAS‐2) tool was used for quality assessment of the included studies [37] which evaluates study quality across four domains: patient selection, index test, reference standard and flow and timing. Each domain is assessed for risk of bias and the first three domains are also assessed for concerns regarding applicability. Alternative tools would have been used for other study types had they been identified in the review (e.g., randomised controlled trials), but for all identified studies the QUADAS‐2 tool was deemed to be the most appropriate.

The review was prospectively registered on the PROPSERO database CRD42023434711 before work on the review started [38].

During the preparation of this work the authors used [Microsoft Copilot] in order to restructure paragraphs and improve readability. After using this tool/service, the authors reviewed and edited the content as needed and take full responsibility for the content of the publication.

## 3 Results

### 3.1 Study Selection

**FIGURE 1:**
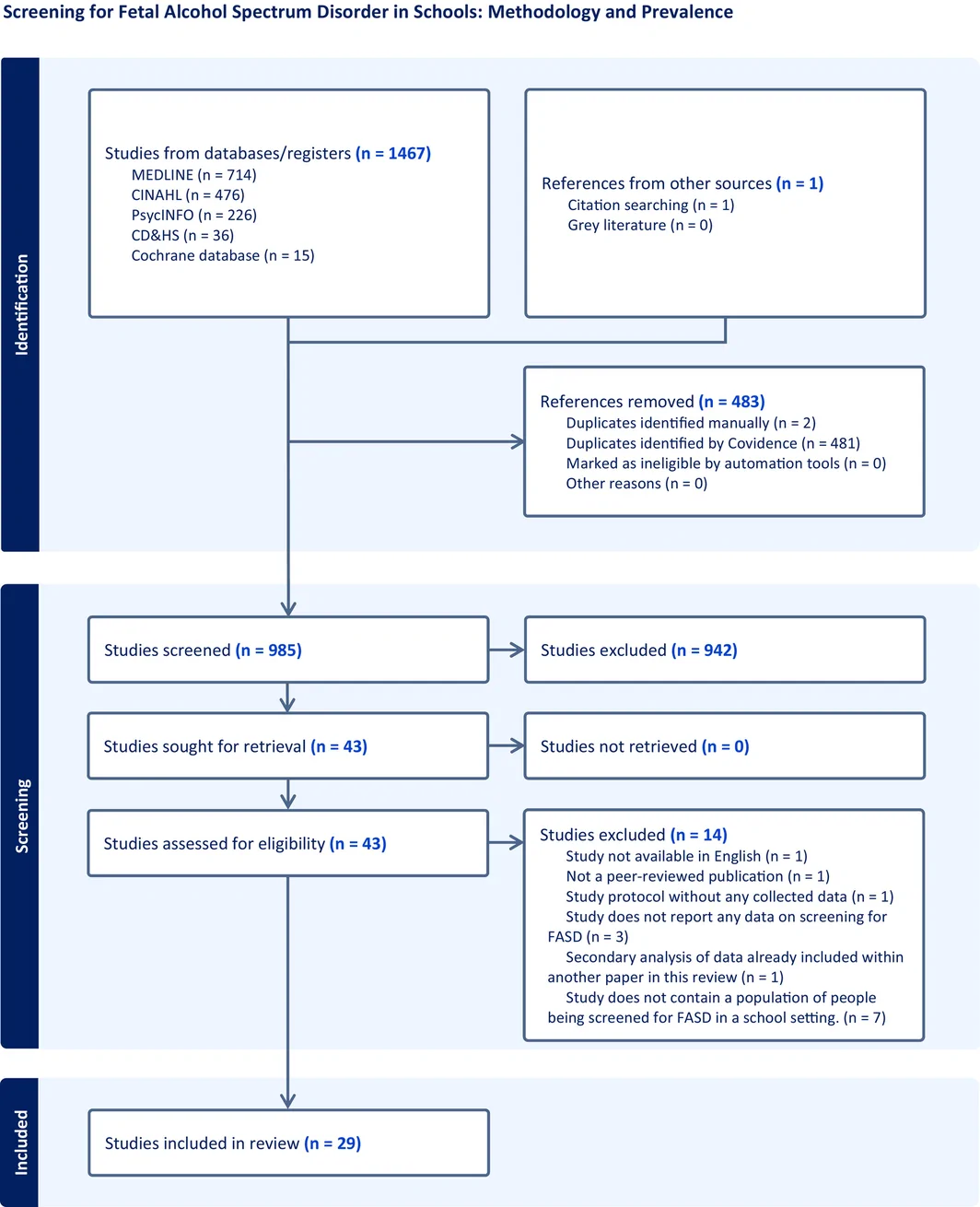
PRISMA 2020 flow diagram illustrating the identification, screening, eligibility assessment and inclusion of studies investigating fetal alcohol spectrum disorder (FASD) screening and prevalence in school settings.

Following the systematic search of databases, 1467 studies were identified from the databases by the search strategy and one additional study was identified by citation searching. After automated deduplication using Covidence, 483 studies were removed and 985 unique references remained. Titles were screened for eligibility and 942 studies were excluded, leaving 43 studies that underwent full‐text screening. The inter‐rater agreement at title and abstract screening was 92.8%, with all agreements resolved by discussion. Fourteen studies were excluded at full‐text screening, leaving 29 studies that were included for data extraction in the review (see Figure 1). The inter‐rater agreement at full‐text screening was 97.6%, with initial disagreement on only one study, which was resolved by discussion. The list of studies excluded at full‐text review, with reasons for exclusion, is given in Appendix S2. Full references, data extraction tables and quality assessment for each included study are given in Appendix S3.

### 3.2 Study Characteristics

#### 3.2.1 Location

A summary of the included studies, including the geographical location the study was conducted in, is given in Table 2. The country with the single largest number of studies was South Africa (*n* = 13), with the remaining split between the USA (*n* = 7), Canada (*n* = 1), the UK (*n* = 1) and four European countries (Croatia *n* = 3, Italy *n* = 2, Poland *n* = 1, Sweden *n* = 1). All studies included children attending mainstream schools, but three made explicit mention of also including pupils with special educational needs and one reported including out‐of‐school children as part of the assessment (even though it was still conducted in a school).

**TABLE 2:** Summary of included studies.

Paper (intext citation)	Study design	Country study conducted in	Number of stages in screening design	Height used as a pre‐screening variable?	Weight used as a pre‐screening variable?	Head size used as a pre‐screening variable?	Parent opt in used as a pre‐screening variables
Chambers [39]	Active case ascertainment	USA	2	Yes	Yes	Yes	No
Clarren [40]	Active case ascertainment	USA	2	Yes	Yes	Yes	Yes
Landgren et al. [41]	Cross‐sectional pilot study design	Sweden	2	Not applicable	Not applicable	Not applicable	Not applicable
Louw et al. [42]	Active case ascertainment	South Africa	3	Yes	Yes	Yes	No
Lubbe [43]	Cohort study	South Africa	3	Yes	Yes	Yes	No
May [44]	Active case ascertainment	Italy	2	Yes	Yes	Yes	No
May et al. [22]	Active case ascertainment	South Africa	2	Yes	Yes	Yes	No
May [45]	Active case ascertainment	Italy	3	Yes	Yes	Yes	No
May [46]	Active case ascertainment	USA	3	Yes	Yes	Yes	No
May [47]	Active case ascertainment	South Africa	3	Yes	Yes	Yes	No
May [47]	Active case ascertainment	South Africa	3	Yes	Yes	Yes	No
May [48]	Active case ascertainment	South Africa	3	Yes	Yes	Yes	No
May [49]	Active case ascertainment	South Africa	2	Yes	Yes	Yes	No
May [50]	Active case ascertainment	USA	3	Yes	Yes	Yes	Yes
May [51]	Active case ascertainment	USA	3	Yes	Yes	Yes	Yes
May [50]	Active case ascertainment	USA	3	Yes	Yes	Yes	No
May [52]	Active case ascertainment	South Africa	3	Yes	Yes	Yes	No
May [52]	Random sample design	USA	2	No	No	No	No
May [53]	Active case ascertainment	South Africa	3	Yes	Yes	Yes	No
McCarthy et al. [54]	Active case ascertainment	UK	2	Yes	Yes	Yes	Yes
Okulicz‐Kozaryn [55]	Active case ascertainment	Poland	2	Yes	Yes	Yes	Yes
Olivier et al. [56]	Active case ascertainment	South Africa	2	Yes	Yes	Yes	No
Petković [57]	Active case ascertainment	Croatia	2	Yes	Yes	Yes	No
Petković [58]	Active case ascertainment	Croatia	2	Yes	Yes	Yes	No
Poitra et al. [59]	Active case ascertainment	Croatia	2	No	No	No	No
Popova et al. [35]	Active case ascertainment	Canada	2	Yes	Yes	Yes	No
Urban [60]	Active case ascertainment	South Africa	2	Yes	Yes	Yes	No
Urban [61]	Active case ascertainment	South Africa	3	Yes	Yes	Yes	No
Viljoen [62]	Active case ascertainment	South Africa	2	Yes	Yes	Yes	No

#### 3.2.2 Study Design

All but three of the studies reported using an active case ascertainment design. All the studies included at least two stages in their screening design, where an initial set of easier‐to‐measure variables was used to identify participants at higher risk of FASD, with those identified then undergoing a more extensive set of assessments. Sixteen studies adopted a 2‐stage design, usually with anthropometric measurements at stage 1 (such as height, weight and head circumference), then a full FAS/FASD assessment, including dysmorphology assessment, as stage 2. Thirteen studies adopted a 3‐stage design, with stage 2 being dysmorphology assessment, with only those found to be positive at this stage going on to a full FAS/FASD assessment.

#### 3.2.3 Co‐Creation, Public Participation and Community Involvement

None of the included studies reported the use of formal co‐creation methods in the design of the research studies. Eight of the studies mentioned the use of some stakeholder input as part of the design process, but the details provided on what this input was and how it impacted on the research design were limited.

#### 3.2.4 Participation Rates

Two studies reported an overall participation rate, 93.6% [42] and 93.7% [56]. Twenty studies reported participation at stage one; these ranged from 16.4% to 98.0%. Eighteen studies reported participation rates at stage 2 ranging from 47.5% to 99.7%. Eight studies reported participation at stage three ranging from 62.3% to 99.4%.

#### 3.2.5 Pre‐Screening Variables

In most studies, the first stage of the process involved the collection of anthropometric measurements (height, weight and head circumference). In some studies, these were the only variables used for which participants moved on to the second age of screening, while in other studies there were additional criteria that could also lead to a child moving on to further stages (Table 3). Three studies did not include any pre‐screening variables, with all children included in the study receiving a dysmorphology assessment [41, 52, 59].

**TABLE 3:** Pre‐screening (stage 1) variables used in included studies.

Variable used for progression to next stage of screening process	Number of studies included in (total *n* = 29)
Low height	26
Low weight	26
Small head circumference	26
School opts child in	9
Parent opts child in	5
Twin of participating child	2
Child is on school's special educational needs register	1
Child is currently or has been previously under the care of the state	1
Child repeating first grade	1
Parents' Evaluation of Developmental Status Tool	1

#### 3.2.6 Child‐Based Assessment Tools Used

A wide range of different child‐based assessment tools were used across the studies (Table 4). In some studies, these tools were explicitly identified as part of the FASD diagnostic process, while in others the tools were completed, but it was not made explicit if or how those results were then used as part of the diagnostic process and it was not possible to differentiate from the published papers. Two studies did not include any child‐based tools as part of their screening process.

**TABLE 4:** Child‐based assessment tools used in included studies.

Tool	Number of studies included (*n* = 29)	Primary brain domain assessed
Ravens Coloured Progressive Matrices	9	Non‐verbal reasoning/fluid intelligence
Test for Reception of Grammar	7	Receptive language
Wechsler Intelligence Scale for Children‐V	6	General intellectual functioning (IQ); attention, working memory and processing speed subdomains
Differential Ability Scales‐II	5	General cognitive ability (IQ)
A Developmental Neuropsychological Assessment	5	Neuropsychological functioning
Bracken School Readiness Assessment	4	School readiness/conceptual knowledge
A Developmental Neuropsychological Assessment‐II	4	Neuropsychological functioning
Beery‐Buktenica Developmental Test of Visual‐Motor Integration	3	Visual‐motor integration
Griffiths Mental Development Scales	3	Global developmental functioning
Rustioni Qualitative Test	2	Language development
Developmental Test of Visual‐Motor Integration	2	Visual‐motor integration
Leiter‐3 Non‐verbal IQ Test	1	Non‐verbal intelligence and attention
Neurobehavioural testing battery (not a specific named instrument)	1	Multiple domains
Fetal Alcohol Syndrome (FAS) Screen	1	FASD‐related neurodevelopmental impairment screening
Wechsler Abbreviated Scale of Intelligence‐II	1	General intellectual functioning (IQ)
Draw‐a‐Person Test	1	Cognitive maturity/non‐verbal conceptual ability
Wechsler Intelligence Scale for Children‐Revised	1	General intellectual functioning (IQ)
Personal Behaviour Checklist	1	Behavioural and emotional functioning
Questionario Osservativo per l Identificazione Precoce delle Difficolta di Apprendimento	1	Learning readiness/early academic skills
Digit Span (Wechsler Intelligence Scale for Children subtest)	1	Working memory and attention
Kaufman Assessment Battery for Children‐II	1	Cognitive processing and learning ability

#### 3.2.7 Case Definition Used for Diagnosis

The majority of studies used some version of the revised Institute of Medicine diagnostic criteria for FASD to inform their case definition, with early studies using a version from 2005 [63] and later studies an updated version from 2016 [8]. Three studies used Canadian FASD guidelines [11], one used a case definition derived from the WHO protocol for prevalence studies and one used an older FAS definition [64]. Papers using the earlier definitions mostly captured cases of full or partial FAS through their screening, rather than all possible categories of FASD.

#### 3.2.8 Dysmorphology

All the included studies except one [47] reported the use of clinical dysmorphology assessment as part of the screening process. Additionally, four of the studies made explicit mention of the use of 2D imaging as part of the dysmorphology assessment, whilst the others did not provide these details.

#### 3.2.9 Exposure Measurement Methodology

Half of the studies (14/29) reported using a maternal lifestyle risk questionnaire to measure levels of PAE. A similar number (13/29) reported using self‐reported maternal history of drinking in pregnancy but did not specify if a structured questionnaire was used. It was unclear whether this represents a genuine methodological difference or simply a difference in the way of reporting the data collection. One study used medical records only to ascertain PAE and one paper did not report the method on PAE measurement.

A structured maternal lifestyle risk questionnaire data about alcohol consumption is collected before, during and after the pregnancy in the context of other lifestyle questions such as diet and exercise in an attempt to reduce social acceptability bias of responses. None of the methods used to collect PAE data reported that they were validated tools.

#### 3.2.10 Quality Assessment

Most studies performed well in the patient selection domain of the QUADAS‐2 checklist, including either the full population in a school or a random subsample of that population. Similarly, most studies were also at low risk of bias in the index test domain, with clearly defined tests and the use of standard validated instruments.

Higher risks of bias were identified in the flow and timing domain, with more between‐study variation. A key difference identified between studies in terms of this quality assessment domain was the way studies dealt with individuals assessed as being negative/low risk in the first stage of the screening process. In some studies, only those identified as being at risk of FASD at this stage went on to future stages of screening and were assessed for a formal diagnosis. Those not referred from the first stage underwent no further testing, meaning potential false negatives at this stage would never have been identified. When estimating their final prevalence rates, these studies tended to assume a prevalence of 0% in this non‐referred group, meaning prevalence rates will be underestimated if there were any false negatives at stage 1.

Other studies, a lower risk of bias on this domain, took a random subsample of those not meeting the stage 1 criteria for further assessment through the rest of the diagnostic pathway, meaning a prevalence estimate could be made for this group as well and adjusted for in the final prevalence estimation. Prevalence rates estimated in this way are likely to be more accurate. The estimates produced from the two methods cannot easily be compared due to the differences in methodology.

The final QUADAS‐2 domain, on the reference standard, had intermediate issues with risks of bias and applicability. This came from two issues: (i) In some studies, the measurements used for pre‐screening or in stage 1 of the study also formed part of the diagnostic reference standard and therefore investigators will have been aware of these earlier results when making a final diagnosis; (ii) As a result of there being no universally agreed definition of FASD and differences between studies in whether they were looking at all FASD or only FAS and alcohol‐related neurodevelopmental disorder, it means the prevalence estimates are often not comparable between studies, as it is not possible to be confident they were estimating the rates for the same underlying condition.

While synthesising the prevalence rates was not the main aim of this paper, our assessment of the methods suggests that the diversity of approaches would make it difficult to meaningfully compare numerical prevalence rates. Indeed, the seminal papers on this topic [19, 65] have been selective, making use of relatively few prevalence estimates to model prevalence estimates for those countries in the world (the vast majority) where prevalence studies are absent or inadequate. It is possible lack of resources impacted the quality of studies; for example, many studies lacked a full assessment of a control group. This may lead to underestimating prevalence due to the assumption that the group not assessed contained no cases of FASD.

## 4 Discussion

Previous reviews have examined prevalence estimates for FASD, including studies carried in mainstream school settings [19], however, methodological differences in the underlying studies have received little attention. The findings of this review shed some light on the differences and similarities in methodology, process and the reporting of those processes. Overall, the studies returned by this review report good practice and low bias for the selected methodology. Generally, these studies have been outcome focused, meaning the key focus of the paper was the prevalence estimate and not the screening methodology; therefore, there is less information on this aspect of each project. There was a wide range of methodology, case definitions and measures used, even though it was apparent that all studies were looking for the same outcome, a clinically significant level of disability caused by prenatal alcohol exposure. Given that school‐based studies represent the most common approach to estimating FASD prevalence in the general population, such variation within a single research setting has important implications for the comparability and interpretation of prevalence estimates. This highlights the need for methodological coherence and cultural adaptability in FASD research to ensure that prevalence estimates are both accurate and comparable.

A range of pre‐screening variables and diagnostic approaches were employed across the different studies identified, with some of the studies reporting no pre‐screening variables. For example, Poitra et al. [59] and the diagnosis relied on clinical dysmorphology assessments based on criteria from Sokol and Clarren [64], with a retrospective application of the criteria from the Institute of Medicine Report on Fetal Alcohol Syndrome [66 ]. In contrast, studies such as Chambers et al. [39] and McCarthy et al. [54] included pre‐screening variables such as height, weight, head circumference and the Parents' Evaluation of Developmental Status. The diagnostic tools used also varied with the approach to assessments, with some studies utilising teacher‐based tools while others relied on parent‐based or child‐based tools. More recently Landgren et al. [41], proposed that both be used.

The differences in methods highlight the lack of international standardisation in FASD screening and the need for validated consistent diagnostic criteria and tools across studies to ensure comparability and reliability of results. However, while an internationally recognised diagnostic code would be helpful, this should not extend to exact specification of the validated tools to be used. There is evidence that psychometric testing should be culturally appropriate [67] and tool selection should remain within the control of local researchers.

Our review found that the majority of the studies undertook an active case ascertainment approach, which is considered the gold standard in establishing the prevalence of FASD. Rates of FASD found varied widely across studies and settings. For instance, Poitra et al. [59] reported a prevalence rate of 0.50% in Croatia, whereas Chambers et al. [39] found a probable FASD rate of 10.9% in a Pacific Southwest city in the USA. The extent to which this is due to differences in study design, population demographics, lifestyle and the rigour of the diagnostic criteria employed, versus the true differences between regions, is unknown. It has been hypothesised that increasing the public awareness of the risks of PAE has inadvertently led to increased misreporting of PAE by mothers in studies such as these [57].

The quality assessment of these studies indicated a generally low risk of bias in patient selection and test conduct. The majority of the studies identified by this review were conducted in mainstream schools (*n* = 28) as opposed to ‘special needs’ schools (*n* = 3). It is likely that the nature of the school setting may have an impact on the prevalence rates obtained. While undertaking the study in mainstream schools gives the advantage of providing a snapshot closer to that of the general population prevalence compared to special needs schools, students with more severe neurodevelopmental impairment associated with FASD may already be educated outside mainstream classrooms, meaning that school‐based prevalence studies conducted primarily in mainstream settings may systematically exclude some affected individuals. There may also be underreporting in mainstream schools due to stigma and lack of awareness of nuanced features of FASD that may be misattributed to behavioural issues.

We found variability in participation rates, which were influenced by factors such as the opt‐in versus opt‐out consent processes and the demographic characteristics of the study populations. For example, Louw et al. [42]. Poitra et al. [59] in Croatia reported a high participation rate of 98% with an opt‐out consent process, while Chambers et al. [39] in the USA had a lower initial participation rate of 36.9% with an opt‐in process. These variations highlight the importance of the consent process design in maximising participation and thus the representativeness of the screening results. Another possible factor in participation rates relates to relative access to services, where studies provide access to referral and support that would otherwise not be available this will likely increase participation [42].

Only five studies allowed parents to opt students into further assessments [40, 50, 51, 54, 58]. This approach could serve as a critical safety net, as some children with FASD may not present with facial features, small stature, or other pre‐screening risk factors but are likely to exhibit challenges at home. The UK study [54] found that all children opted in by parents exhibited significant neurodevelopmental deficits, even if not all were diagnosed with FASD. This suggests that incorporating parent opt‐in mechanisms in this type of study could enhance case identification.

### 4.1 Outcome Measures

The field of FASD is relatively new and has evolved with changes to the terminology reflecting progressive increase in understanding, expertise and evidence. This means that earlier papers are more likely to use case definitions in line with the more specific diagnosis of FAS, which represents the most recognisable end of the FASD spectrum, characterised by distinctive facial features. However, people living with FASD without facial features can experience neurodevelopmental impacts that are comparable in severity [68]. Moreover, such individuals can be at greater disadvantage due to the increased risk of their condition going undiagnosed. It is therefore just as important to include these groups in prevalence studies.

A lack of robust prevalence data is a barrier to adequate funding for prevention, diagnosis and support capacity [17]. While clinicians may claim one diagnostic code has merit over another, from a research and epidemiological perspective there is value in an internationally agreed guideline regardless of its exact nature, as this would allow more accurate synthesis and more robust repeatability assessment of numerical data [69].

### 4.2 Reporting of Community Engagement

The review revealed a gap in reporting on co‐creation and collaboration with communities and those with lived experience on study design. Although co‐creation is considered beneficial [28], none of the identified studies explicitly reported this. Only six studies referred to involving the public or those with lived experience in some way [42, 54, 56, 57, 58, 70]. Given the sensitive nature of FASD research, it is likely that co‐creation and community engagement occurred with more studies than were not reported.

The papers report the findings of complex, often multi‐phase, research studies. This causes limitations in the detail that can be reported when writing up, since authors must comply with the word limit of peer reviewed journals. Publishing separate papers linked to the methodology and implementation of studies is one possible way to address this. For example, the team which undertook the UK prevalence study has since published the findings from interviews with parents who took part [31, 71].

### 4.3 Exposure Measures

PAE was measured by most studies with either a maternal lifestyle risk questionnaire or self‐reported maternal history of PAE. None reported use of biomarkers such as blood alcohol level or meconium samples. This is because at the age at which active case ascertainment for FASD becomes optimum (approximately 8 years of age) these more objective measures are no longer possible retrospectively. Self‐reported PAE is susceptible to both recall bias and social desirability bias. Recall bias refers to the declining ability to accurately remember alcohol consumption over time. Social desirability bias arises from the influence of perceived societal judgement, which may lead individuals to underreport their alcohol use. Studies have shown individuals forget details such as the time, frequency, quantity or type of alcohol consumed anden when interviewed alongside blood alcohol testing, PAE was significantly underreported [72].

### 4.4 Strengths and Limitations

The analysis presented in this paper was limited to a descriptive synthesis, lacking the contextual insights offered by qualitative findings. It is possible that more contextual information could be published in companion papers, as is the case for the UK study [6, 54, 71]. Such studies could add useful contextual information (and could contribute to our aim of assessing cocreation and community engagement) but will have been excluded using our review protocol. As this review did not formally examine associations between methodological characteristics and prevalence estimates, interpretations regarding sources of variability should be considered exploratory. This review was limited to studies published in English. As a result, relevant studies published in other languages would not have been captured, thus limiting representation from non‐English‐speaking regions.

### 4.5 Implications for Further Research

Further exploration into methodology and approaches used when looking for FASD in schools is needed, beyond that which is reported in the peer reviewed literature. This review deliberately focused on school settings, as schools provide access to large, age‐defined cohorts and represent the most common and feasible context for identifying FASD in the general population of children. Active case ascertainment studies are complex and in the case of FASD, relate to a sensitive subject with unique ethical concerns. It is unrealistic to expect the full extent of the task to be reflected in necessarily brief reports of a prevalence study. With increasing emphasis on transparency and reproducibility of research methods, further research is needed to fully explore the process of these studies to inform future measurement of prevalence for common, but under‐diagnosed conditions such as FASD.

## 5 Conclusion

This systematic review highlights the complexities and variability in methodologies employed in studies investigating FASD prevalence in school settings. The studies demonstrated good practices and a low risk of bias. There were significant differences in pre‐screening variables and case definitions, highlighting the lack of standardisation in this area. These methodological inconsistencies may contribute to discrepancies in reported prevalence rates and limit the comparability of findings across studies.

The findings of this review support the need for an internationally recognised diagnostic framework to enhance consistency while allowing flexibility for culturally appropriate and locally adaptable tools. The inclusion of parent opt‐in mechanisms and a stronger focus on community engagement and co‐creation could improve case identification and participation rates further.

The findings of this review also support the utilisation of opt‐out consent processes to improve participation rates, of a control group to check sensitivity of the pre‐screening method used and allowing parents to opt their child in to further assessment when this is not triggered by pre‐screening.

Ultimately, this review highlights that robust and transparent methodologies are important for obtaining accurate prevalence data, which is critical for securing adequate resources for prevention, diagnosis and support services. Future research should prioritise the development of standardised, reproducible methods and a research guideline while acknowledging the ethical and practical challenges unique to FASD prevalence studies. Addressing these gaps will provide a clearer understanding of the true global and regional burden of FASD and ensure that affected individuals receive the support they need.

## Author Contributions

Each author certifies that their contribution to this work meets the standards of the International Committee of Medical Journal Editors.

## Funding

The authors have nothing to report.

## Conflicts of Interest

The authors declare no conflicts of interest.

## Supporting information


**Appendix S1:** Studies excluded at full‐text review.

**Data S1:** Supporting Information.

**Data S2:** Supporting Information.

## Data Availability

The data that supports the findings of this study are available in the Supporting Information of this article.
