# Supplementary material for: Fetal Alcohol Spectrum Disorder Prevalence Studies in School Settings: A Global Systematic Review of Methods, Measures, Strengths and Limitations
Source: Drug Alcohol Rev. 2026 Sep 10;45(6):e70233. doi: 10.1111/dar.70233 (PMC13559213; doi:10.1111/dar.70233)
Supplement: Supplementary file 1 [file DAR-45-0-s001.docx]

# 1. Chambers 2019

Chambers, Christina D; Coles, Claire; Kable, Julie; Akshoomoff, Natacha; Xu, Ronghui; Zellner, Jennifer A; Honerkamp-Smith, Gordon; Manning, Melanie A; Adam, Margaret P; Jones, Kenneth Lyons. Fetal Alcohol Spectrum Disorders in a Pacific Southwest City: Maternal and Child Characteristics. Alcoholism, clinical and experimental research 2019;43(12):2578-2590

**Study design**

Active case ascertainment

**Country study conducted in**

USA

**Was there cocreation of the design of the research study by people with lived experience of FASD?**

Not reported

**Number of stages in screening design**

2 stages

**Consent process**

Opt in

**Pre-screening variables**

- Height
- Weight
- Head circumference
- PEDS (Parents' Evaluation of Developmental Status)
- Child repeating first grade

**Study exclusion criteria**

None reported

**Participation rates**

Stage 1: 36.9% (1,628/4,409)

Stage 2: 77.2% (927/1,201)

**Non-participation data**

Non-participants either did not respond to invitations, or declined to consent to screening.

**Type of school provision**

Mainstream

**Sample demographic categories collected (including both those who did and did not participate in the study)**

None

**Participant demographic categories collected (including just those who did participate in the study)**

- Maternal age
- Maternal education
- Maternal ethnicity
- Household income
- Child age
- Child sex

**Did the study use a pre-determined definition of an FASD case?**

Yes

**Guideline used for FASD case definition**

Other: Consensus criteria for FASD developed for the CoFASP study (Hoyme, 2016)

**Measurements used: Dysmorphology**

- Clinical dysmorphology assessment
- 2D imaging

**Measurements used: Parent based tools**

- CBCL: Child Behavior Checklist

**Measurements used: Child based tools**

- BBCS: Bracken Basic Concept Scale
- DAS II: Differential Ability Scales
- NEPSY: Developmental Neuropsychological Assessment
- VABS: Vineland Adaptive Behavior Scale
- VMI: Visual-Motor Integration

**Measurements used: Genetic analysis**

None

**Measurements used: Teacher based tools**

- TRF: Teacher Report Form

**Children identified as cases seen by a clinician?**

Yes

**Prenatal alcohol exposure data measured**

- Self-reported maternal history

**Prenatal drug exposure data measured**

- Illicit drugs
- Other prescription drugs
- Other non-prescription drugs
- Tobacco

**People individual child results shared with**

Not reported

**Estimated FASD rates**

1.9-2.3% (reported as a “minimum prevalence” estimate)

**Quality Assessment (QUANDAS-2)**

Patient selection

Was a consecutive or random sample of patients enrolled? Yes

Was a case-control design avoided? Yes

Did the study avoid inappropriate exclusions? Yes

Overall - Could the selection of patient have introduced bias? Level of risk: Low

Applicability - Is there concern that the included patients do not match the review question? Level of risk: Low

Index test(s)

Were the index test results interpreted without knowledge of the results of the reference standard? Yes

If a threshold was used, was it pre-specified? Yes

Overall - Could the conduct or interpretation of the index test have introduced bias? Level of risk: Low

Applicability - Is there concern that the index test, its conduct, or interpretation differ from the review question? Level of risk: Low

Reference standard

Is the reference standard likely to correctly classify the target condition? Unclear - No definitive FASD diagnostic test

Were the reference standard results interpreted without knowledge of the results of the index test? No - Reference standard includes index tests

Overall - Could the reference standard, its conduct, or its interpretation have introduced bias? Level of risk: Unclear - No definitive FASD diagnostic test

Applicability - Is there concern that the target condition as defined by the reference standard does not match the review question? Level of risk: Unclear - No definitive FASD diagnostic test

Flow and timing

Was there an appropriate interval between index test(s) and reference standard? Yes

Did all patients receive a reference standard? Yes

Did patients receive the same reference standard? Yes

Were all patients included in the analysis? Yes

Overall - Could the patient flow have introduced bias? Level of risk: Low

# 2. Clarren 2001

Clarren, S K; Randels, S P; Sanderson, M; Fineman, R M. Screening for fetal alcohol syndrome in primary schools: a feasibility study. Teratology 2001;63(1):3-10

**Study design**

Active case ascertainment

**Country study conducted in**

USA

**Was there cocreation of the design of the research study by people with lived experience of FASD?**

Not reported

**Number of stages in screening design**

2 stages

**Consent process**

Not reported

**Pre-screening variables**

- Height
- Weight
- Head circumference
- School opts child in
- Parent opts child in

**Study exclusion criteria**

None reported

**Participation rates**

Stage 1: 38.8% (3,740/9,630)

Stage 2: 47.5% (29/61)

**Non-participation data**

Most missing data either came from schools choosing not to participate in the study, or parents not giving consent for screening.

**Type of school provision**

- Mainstream
- Special educational needs

**Sample demographic categories collected (including both those who did and did not participate in the study)**

None

**Participant demographic categories collected (including just those who did participate in the study)**

- Maternal ethnicity

**Did the study use a pre-determined definition of an FASD case?**

Yes

**Guideline used for FASD case definition**

Canadian; Other: Astley & Clarren, 2000

**Measurements used: Dysmorphology**

Clinical dysmorphology assessment

**Measurements used: Parent based tools (validation status)**

Not reported

**Measurements used: Child based tools (validation status)**

Not reported

**Measurements used: Genetic analysis**

Not reported

**Measurements used: Teacher based tools (validation status)**

Not reported

**Children identified as cases seen by a clinician?**

No

**Prenatal alcohol exposure data measured**

- Self-reported maternal history

**Prenatal drug exposure data measured**

Not reported

**People individual child results shared with**

- Parents/carers

**Estimated FASD rates**

0.31% (reported as a “minimal prevalence” estimate)

**Quality Assessment (QUANDAS-2)**

Patient selection

Was a consecutive or random sample of patients enrolled? Yes

Was a case-control design avoided? Yes

Did the study avoid inappropriate exclusions? Yes

Overall - Could the selection of patient have introduced bias? Level of risk: Low

Applicability - Is there concern that the included patients do not match the review question? Level of risk: Low

Index test(s)

Were the index test results interpreted without knowledge of the results of the reference standard? Unclear

If a threshold was used, was it pre-specified? Yes

Overall - Could the conduct or interpretation of the index test have introduced bias? Level of risk: Unclear

Applicability - Is there concern that the index test, its conduct, or interpretation differ from the review question? Level of risk: Low

Reference standard

Is the reference standard likely to correctly classify the target condition? No - This study looked for full FAS only

Were the reference standard results interpreted without knowledge of the results of the index test? Unclear

Overall - Could the reference standard, its conduct, or its interpretation have introduced bias? Level of risk: Low

Applicability - Is there concern that the target condition as defined by the reference standard does not match the review question? Level of risk: High - This study looked for full FAS only

Flow and timing

Was there an appropriate interval between index test(s) and reference standard? Yes

Did all patients receive a reference standard? No – only those identified at stage 1 were given full testing at stage 2

Did patients receive the same reference standard? Yes

Were all patients included in the analysis? Yes – those not referred from stage 1 were assumed to not have FAS

Overall - Could the patient flow have introduced bias? Level of risk: Unclear – prevalence may be underestimated due to FAS cases not being picked up in the stage 1 screening

# 3. Landgren 2025

Landgren, V., Knez, R., Karlsson, D., Fernmo, S., Johnson, M., Svensson, L., & Landgren, M. (2025). Prevalence of Fetal Alcohol Spectrum Disorders: A Pilot Study in Western Sweden. *Acta Paediatrica*.

**Study design**

Cross‑sectional pilot study design

**Country study conducted in**

Sweden

**Was there cocreation of the design of the research study by people with lived experience of FASD?**

No co-creation, some stakeholder involvement with school communities

**Number of stages in screening design**

2 stages

**Consent process**

Opt in

**Pre-screening variables**

Not applicable

**Study exclusion criteria**

None reported

**Participation rates**

64%

**Non-participation data**

None reported

**Type of school provision**

Mainstream

**Sample demographic categories collected (including both those who did and did not participate in the study)**

None reported

**Participant demographic categories collected (including just those who did participate in the study)**

- Not reported

**Did the study use a pre-determined definition of an FASD case?**

Yes

**Guideline used for FASD case definition**

Other: IOM Hoyme et al

**Measurements used: Dysmorphology**

Clinical dysmorphology assessment in line with Hoyme et al (IQR)

**Measurements used: Parent based tools (validation status)**

None

**Measurements used: Child based tools (validation status)**

Leiter-III non-verbal IQ test (validated) and performance ratings (unclear)

**Measurements used: Genetic analysis**

None

**Measurements used: Teacher based tools (validation status)**

ESSENCE-questionnaire (validated)

Strengths and difficulties questionnaire SDQ (Validated)

**Children identified as cases seen by a clinician?**

Yes

**Prenatal alcohol exposure data measured**

- Maternal Risk Questionnaire

**Prenatal drug exposure data measured**

Not reported

**People individual child results shared with**

Not reported

**Estimated FASD rates**

5.5% (95% CI 3.3-8.4) (combining FAS and pFAS and ARND)

**Quality Assessment (QUANDAS-2)**

Patient selection

Was a consecutive or random sample of patients enrolled? Yes

Was a case-control design avoided? Yes

Did the study avoid inappropriate exclusions? Yes

Overall - Could the selection of patient have introduced bias? Level of risk: Low

Applicability - Is there concern that the included patients do not match the review question? Level of risk: Low

Index test(s)

Were the index test results interpreted without knowledge of the results of the reference standard? Yes

If a threshold was used, was it pre-specified? Yes

Overall - Could the conduct or interpretation of the index test have introduced bias? Level of risk: Low

Applicability - Is there concern that the index test, its conduct, or interpretation differ from the review question? Level of risk: Low

Reference standard

Is the reference standard likely to correctly classify the target condition? YES

Were the reference standard results interpreted without knowledge of the results of the index test? Yes

Overall - Could the reference standard, its conduct, or its interpretation have introduced bias? Level of risk: Low

Applicability - Is there concern that the target condition as defined by the reference standard does not match the review question? Level of risk: Low

Flow and timing

Was there an appropriate interval between index test(s) and reference standard? Yes

Did all patients receive a reference standard? YES

Did patients receive the same reference standard? Yes

Were all patients included in the analysis? Yes

Overall - Could the patient flow have introduced bias? Level of risk: Low

# 4. Louw 2024

Louw, J. G., Broodryk, M., White, L., Acker, D., Viljoen, D. L., & Olivier, L. (2024). A multi‐year, multi‐site study of the prevalence of fetal alcohol syndrome in South Africa. *Alcohol: Clinical and Experimental Research*, *48*(5), 867-879.

**Study design**

Active case ascertainment

**Country study conducted in**

South Africa

**Was there cocreation of the design of the research study by people with lived experience of FASD?**

No co-creation, some stakeholder involvement

**Number of stages in screening design**

3 stages

**Consent process**

Opt in

**Pre-screening variables**

- Height
- Weight
- Head circumference

**Study exclusion criteria**

None reported

**Participation rates**

Overall: Of eligible that were consented in: 93.7%

**Non-participation data**

None reported

**Type of school provision**

Mainstream

**Sample demographic categories collected (including both those who did and did not participate in the study)**

None

**Participant demographic categories collected (including just those who did participate in the study)**

- Maternal age
- Child age
- Child sex
- Birth order

**Did the study use a pre-determined definition of an FASD case?**

Yes

**Guideline used for FASD case definition**

Other: Gestalt approach (informed by Hoyme et all)

**Measurements used: Dysmorphology**

Clinical dysmorphology assessment

**Measurements used: Parent based tools (validation status)**

None

**Measurements used: Child based tools (validation status)**

NEPSY II (Validated)

**Measurements used: Genetic analysis**

None

**Measurements used: Teacher based tools (validation status)**

None

**Children identified as cases seen by a clinician?**

Yes

**Prenatal alcohol exposure data measured**

- Maternal Risk Questionnaire

**Prenatal drug exposure data measured**

- Tobacco and other drug

**People individual child results shared with**

School, Family Doctor

**Estimated FASD rates**

8.02% FASD (all)

6.30% Full FAS

**Quality Assessment (QUANDAS-2)**

Patient selection

Was a consecutive or random sample of patients enrolled? Yes

Was a case-control design avoided? Yes

Did the study avoid inappropriate exclusions? Yes

Overall - Could the selection of patient have introduced bias? Level of risk: Low

Applicability - Is there concern that the included patients do not match the review question? Level of risk: Low

Index test(s)

Were the index test results interpreted without knowledge of the results of the reference standard? Yes

If a threshold was used, was it pre-specified? Yes

Overall - Could the conduct or interpretation of the index test have introduced bias? Level of risk: Low

Applicability - Is there concern that the index test, its conduct, or interpretation differ from the review question? Level of risk: Low

Reference standard

Is the reference standard likely to correctly classify the target condition? YES

Were the reference standard results interpreted without knowledge of the results of the index test? Yes

Overall - Could the reference standard, its conduct, or its interpretation have introduced bias? Level of risk: Low

Applicability - Is there concern that the target condition as defined by the reference standard does not match the review question? Level of risk: Low

Flow and timing

Was there an appropriate interval between index test(s) and reference standard? Yes

Did all patients receive a reference standard? No – ACA design

Did patients receive the same reference standard? Yes

Were all patients included in the analysis? Yes

Overall - Could the patient flow have introduced bias? Level of risk: Low

# 5. Lubbe 2017

Lubbe, Melissa; van Walbeek, Corné; Vellios, Nicole. The Prevalence of Fetal Alcohol Syndrome and Its Impact on a Child's Classroom Performance: A Case Study of a Rural South African School. International journal of environmental research and public health 08/09/ 2017;14(8):

**Study design**

Cohort study

**Country study conducted in**

South Africa

**Was there cocreation of the design of the research study by people with lived experience of FASD?**

Not reported

**Number of stages in screening design**

3 stages

**Consent process**

Opt out

**Pre-screening variables**

- Height
- Weight
- Head circumference

**Study exclusion criteria**

None reported

**Participation rates**

At third stage: 165/166 = 99.4%

**Non-participation data**

Not reported

**Type of school provision**

Mainstream

**Sample demographic categories collected (including both those who did and did not participate in the study)**

- Child age
- Child sex
- Deprivation level of school area

**Participant demographic categories collected (including just those who did participate in the study)**

- Child age
- Child sex

**Did the study use a pre-determined definition of an FASD case?**

Yes

**Guideline used for FASD case definition**

Other: Hoyme 2016

**Measurements used: Dysmorphology**

Clinical dysmorphology assessment

**Measurements used: Parent based tools (validation status)**

None

**Measurements used: Child based tools (validation status)**

None

**Measurements used: Genetic analysis**

None

**Measurements used: Teacher based tools (validation status)**

None

**Children identified as cases seen by a clinician?**

Yes

**Prenatal alcohol exposure data measured**

- Medical record

**Prenatal drug exposure data measured**

Not reported

**People individual child results shared with**

Not reported

**Estimated FASD rates**

Probable FAS: 12.7%

Possible FAS: 18.7%

**Quality Assessment (QUANDAS-2)**

Patient selection

Was a consecutive or random sample of patients enrolled? Yes

Was a case-control design avoided? Yes

Did the study avoid inappropriate exclusions? Yes

Overall - Could the selection of patient have introduced bias? Level of risk: Low

Applicability - Is there concern that the included patients do not match the review question? Level of risk: Low

Index test(s)

Were the index test results interpreted without knowledge of the results of the reference standard? Yes

If a threshold was used, was it pre-specified? Yes

Overall - Could the conduct or interpretation of the index test have introduced bias? Level of risk: Low

Applicability - Is there concern that the index test, its conduct, or interpretation differ from the review question? Level of risk: Low

Reference standard

Is the reference standard likely to correctly classify the target condition? Yes

Were the reference standard results interpreted without knowledge of the results of the index test? Unclear - Doctor would have known reason for referral although only higher risk children referred

Overall - Could the reference standard, its conduct, or its interpretation have introduced bias? Level of risk: Low

Applicability - Is there concern that the target condition as defined by the reference standard does not match the review question? Level of risk: Low

Flow and timing

Was there an appropriate interval between index test(s) and reference standard? Yes

Did all patients receive a reference standard? No

Did patients receive the same reference standard? No - Study design

Were all patients included in the analysis? Yes

Overall - Could the patient flow have introduced bias? Level of risk: Low

# 6. May 2006

May, Philip A; Fiorentino, Daniela; Phillip Gossage, J; Kalberg, Wendy O; Eugene Hoyme, H; Robinson, Luther K; Coriale, Giovanna; Jones, Kenneth Lyons; del Campo, Miguel; Tarani, Luigi; Romeo, Marina; Kodituwakku, Piyadasa W; Deiana, Luca; Buckley, David; Ceccanti, Mauro. Epidemiology of FASD in a province in Italy: Prevalence and characteristics of children in a random sample of schools. Alcoholism, clinical and experimental research. 2006;30(9):1562-1575

**Study design**

Active case ascertainment

**Country study conducted in**

Italy

**Was there cocreation of the design of the research study by people with lived experience of FASD?**

Not reported

**Number of stages in screening design**

2 stages

**Consent process**

Opt in

**Pre-screening variables**

- Height
- Weight
- Head circumference
- School opts child in

**Study exclusion criteria**

None reported

**Participation rates**

Consent for stage 1: 50.0% (543/1086)
Participation in stage 2: 98.7% (230/233)

**Non-participation data**

No information provided beyond lack of consent.

**Type of school provision**

Mainstream

**Sample demographic categories collected (including both those who did and did not participate in the study)**

- Maternal education

**Participant demographic categories collected (including just those who did participate in the study)**

- Maternal age
- Child age
- Child sex

**Did the study use a pre-determined definition of an FASD case?**

Yes

**Guideline used for FASD case definition**

IOM diagnostic criteria

**Measurements used: Dysmorphology**

Clinical dysmorphology assessment

**Measurements used: Parent based tools (validation status)**

- Pelham Disruptive Behaviors Disorder (DBD) Scale

**Measurements used: Child based tools (validation status)**

- Ravens Coloured Progressive Matrices
- Rustioni Qualitative Test

**Measurements used: Genetic analysis**

None

**Measurements used: Teacher based tools (validation status)**

- Personal Behavior Checklist (PBCL)

**Children identified as cases seen by a clinician?**

No

**Prenatal alcohol exposure data measured**

- Self-reported maternal history

**Prenatal drug exposure data measured**

- Tobacco

**People individual child results shared with**

Not reported

**Estimated FASD rates**

Probable FASD: 3.5%

**Quality Assessment (QUANDAS-2)**

Patient selection

Was a consecutive or random sample of patients enrolled? Yes

Was a case-control design avoided? Yes

Did the study avoid inappropriate exclusions? Yes

Overall - Could the selection of patient have introduced bias? Level of risk: Low

Applicability - Is there concern that the included patients do not match the review question? Level of risk: Low

Index test(s)

Were the index test results interpreted without knowledge of the results of the reference standard? Yes

If a threshold was used, was it pre-specified? Yes

Overall - Could the conduct or interpretation of the index test have introduced bias? Level of risk: Low

Applicability - Is there concern that the index test, its conduct, or interpretation differ from the review question? Level of risk: Low

Reference standard

Is the reference standard likely to correctly classify the target condition? Unclear - No agreed diagnostic criteria for FASD

Were the reference standard results interpreted without knowledge of the results of the index test? No - Index tests form part of the reference standard

Overall - Could the reference standard, its conduct, or its interpretation have introduced bias? Level of risk: Unclear - No agreed diagnostic criteria for FASD

Applicability - Is there concern that the target condition as defined by the reference standard does not match the review question? Level of risk: Unclear - No agreed diagnostic criteria for FASD

Flow and timing

Was there an appropriate interval between index test(s) and reference standard? Yes

Did all patients receive a reference standard? No - Only those with suspected FASD, plus a random subsample

Did patients receive the same reference standard? Yes

Were all patients included in the analysis? Yes

Overall - Could the patient flow have introduced bias? Level of risk: Low - Although not all are given the reference standard, an estimate is produced incorporating the random subsample, which helps to mitigate bias.

# 7. May 2007

May, Philip A; Gossage, J Phillip; Marais, Anna-Susan; Adnams, Colleen M; Hoyme, H Eugene; Jones, Kenneth L; Robinson, Luther K; Khaole, Nathaniel C O; Snell, Cudore; Kalberg, Wendy O; Hendricks, Loretta; Brooke, Lesley; Stellavato, Chandra; Viljoen, Denis L. The epidemiology of fetal alcohol syndrome and partial FAS in a South African community. Drug and alcohol dependence 2007;88(2-3):259-271

**Study design**

Active case ascertainment

**Country study conducted in**

South Africa

**Was there cocreation of the design of the research study by people with lived experience of FASD?**

Not reported

**Number of stages in screening design**

2 stages

**Consent process**

- Opt out at stage 1
- Opt in at stage 2

**Pre-screening variables**

- Height
- Weight
- Head circumference

**Study exclusion criteria**

None reported

**Participation rates**

Stage 1: 80.8% (818/1013)

Stage 2: 95.5% (236/247)

**Non-participation data**

None reported

**Type of school provision**

Mainstream

**Sample demographic categories collected (including both those who did and did not participate in the study)**

None

**Participant demographic categories collected (including just those who did participate in the study)**

- Maternal age
- Maternal education
- Maternal ethnicity
- Household income
- Child age
- Child sex

**Did the study use a pre-determined definition of an FASD case?**

Yes

**Guideline used for FASD case definition**

Other: Hoyme et al, 2005

**Measurements used: Dysmorphology**

Clinical dysmorphology assessment

**Measurements used: Parent based tools (validation status)**

- Personal Behaviours Checklist

**Measurements used: Child based tools (validation status)**

- Tests of Reception Grammar
- Ravens Coloured Progressive Matrices

**Measurements used: Genetic analysis**

None

**Measurements used: Teacher based tools (validation status)**

None

**Children identified as cases seen by a clinician?**

Yes

**Prenatal alcohol exposure data measured**

- Maternal Risk Questionnaire

**Prenatal drug exposure data measured**

- Illicit drugs
- Tobacco

**People individual child results shared with**

Not reported

**Estimated FASD rates**

6.80% (reported as a “conservative in-school prevalence)

**Quality Assessment (QUANDAS-2)**

Patient selection

Was a consecutive or random sample of patients enrolled? Yes

Was a case-control design avoided? Yes

Did the study avoid inappropriate exclusions? Yes

Overall - Could the selection of patient have introduced bias? Level of risk: Low

Applicability - Is there concern that the included patients do not match the review question? Level of risk: Low

Index test(s)

Were the index test results interpreted without knowledge of the results of the reference standard? Yes

If a threshold was used, was it pre-specified? Yes

Overall - Could the conduct or interpretation of the index test have introduced bias? Level of risk: Low

Applicability - Is there concern that the index test, its conduct, or interpretation differ from the review question? Level of risk: Low

Reference standard

Is the reference standard likely to correctly classify the target condition? Yes

Were the reference standard results interpreted without knowledge of the results of the index test? Yes

Overall - Could the reference standard, its conduct, or its interpretation have introduced bias? Level of risk: Low

Applicability - Is there concern that the target condition as defined by the reference standard does not match the review question? Level of risk: Low

Flow and timing

Was there an appropriate interval between index test(s) and reference standard? Yes

Did all patients receive a reference standard? No - Only those suspected of FASD, plus random controls

Did patients receive the same reference standard? Yes

Were all patients included in the analysis? Yes

Overall - Could the patient flow have introduced bias? Level of risk: Low - Although not all participants were included, applying the refence standard to a random subsample should mitigate the risk of bias

# 8. May 2011

May, Philip A; Fiorentino, Daniela; Coriale, Giovanna; Kalberg, Wendy O; Hoyme, H Eugene; Aragón, Alfredo S; Buckley, David; Stellavato, Chandra; Gossage, J Phillip; Robinson, Luther K; Jones, Kenneth Lyons; Manning, Melanie; Ceccanti, Mauro. Prevalence of children with severe fetal alcohol spectrum disorders in communities near Rome, Italy: new estimated rates are higher than previous estimates. International journal of environmental research and public health 2011;8(6):2331-2351

**Study design**

Active case ascertainment

**Country study conducted in**

Italy

**Was there cocreation of the design of the research study by people with lived experience of FASD?**

Not reported

**Number of stages in screening design**

3 stages

**Consent process**

Opt in

**Pre-screening variables**

- Height
- Weight
- Head circumference
- School opts child in

**Study exclusion criteria**

None reported

**Participation rates**

Consent at stage 1: 49.1% (976/1988)
Participation at stage 2: All eligible children participated in stage 2

**Non-participation data**

No information provided other than that those excluded did not consent to participate

**Type of school provision**

Mainstream

**Sample demographic categories collected (including both those who did and did not participate in the study)**

None

**Participant demographic categories collected (including just those who did participate in the study)**

- Maternal age
- Maternal education
- Child age
- Child sex

**Did the study use a pre-determined definition of an FASD case?**

Yes

**Guideline used for FASD case definition**

Other: IOM diagnostic criteria

**Measurements used: Dysmorphology**

Clinical dysmorphology assessment

**Measurements used: Parent based tools (validation status)**

- Pelham Disruptive Behaviors Disorder (DBD) Scale

**Measurements used: Child based tools (validation status)**

- Ravens Coloured Progressive Matrices
- Rustioni Qualitative Test
- Wechsler Intelligence Scale for Children Revised (WISC-R)
- Personal Behavior Checklist (PBCL)
- Questionario Osservativo per Iâ Identificazione Precoce delle Difficult di Aprendimento (IPDA)

**Measurements used: Genetic analysis**

None

**Measurements used: Teacher based tools (validation status)**

- Teacher Report form

**Children identified as cases seen by a clinician?**

No

**Prenatal alcohol exposure data measured**

- Self-reported maternal history

**Prenatal drug exposure data measured**

- Tobacco

**People individual child results shared with**

None reported

**Estimated FASD rates**

5.90%: 95% CI (2.3%, 6.3%)

**Quality Assessment (QUANDAS-2)**

Patient selection

Was a consecutive or random sample of patients enrolled? Yes

Was a case-control design avoided? Yes

Did the study avoid inappropriate exclusions? Yes

Overall - Could the selection of patient have introduced bias? Level of risk: Low

Applicability - Is there concern that the included patients do not match the review question? Level of risk: Low

Index test(s)

Were the index test results interpreted without knowledge of the results of the reference standard? Yes

If a threshold was used, was it pre-specified? Yes

Overall - Could the conduct or interpretation of the index test have introduced bias? Level of risk: Low

Applicability - Is there concern that the index test, its conduct, or interpretation differ from the review question? Level of risk: Low

Reference standard

Is the reference standard likely to correctly classify the target condition? Unclear - No standard FASD diagnostic criteria

Were the reference standard results interpreted without knowledge of the results of the index test? No - Reference standard includes index tests

Overall - Could the reference standard, its conduct, or its interpretation have introduced bias? Level of risk: Unclear - No standard FASD diagnostic criteria

Applicability - Is there concern that the target condition as defined by the reference standard does not match the review question? Level of risk: Unclear - No standard FASD diagnostic criteria

Flow and timing

Was there an appropriate interval between index test(s) and reference standard? Yes

Did all patients receive a reference standard? No - Only those suspected of FASD, plus random controls

Did patients receive the same reference standard? Yes

Were all patients included in the analysis? Yes

Overall - Could the patient flow have introduced bias? Level of risk: Low - Although not all participants were included, applying the refence standard to a random subsample should mitigate the risk of bias

# 9. May 2014

May, Philip A; Baete, Amy; Russo, Jaymi; Elliott, Amy J; Blankenship, Jason; Kalberg, Wendy O; Buckley, David; Brooks, Marita; Hasken, Julie; Abdul-Rahman, Omar; Adam, Margaret P; Robinson, Luther K; Manning, Melanie; Hoyme, H Eugene. Prevalence and characteristics of fetal alcohol spectrum disorders. Pediatrics 2014;134(5):855-866

**Study design**

Active case ascertainment

**Country study conducted in**

USA

**Was there cocreation of the design of the research study by people with lived experience of FASD?**

Not reported

**Number of stages in screening design**

3 stages

**Consent process**

Opt in

**Pre-screening variables**

- Height
- Weight
- Head circumference
- School opts child in

**Study exclusion criteria**

None reported

**Participation rates**

Consent for stage 1: 70.5% (1433/2033)

Consent for stage 2: 71.1% (512/720)

Consent for stage 3 (child assessment): 65% (187/287)

Consent for stage 3 (maternal interview): 53% (153/287)

**Non-participation data**

Of the 600 who did not consent at stage 1, 112 refused consent and 488 did not return the consent form.

Of the 208 who did not consent at stage 2, 9 refused consent, 196 did not return the consent form, and 3 dropped out of the study/moved to a different area.

Of the 100 who did not consent to child assessment at stage 3, 16 refused testing, 61 were not possible to contact, 13 did not show up for testing, 9 could not be tested due to language barriers, and in 1 child testing was incomplete.

Of the 134 who did not consent to a maternal interview at stage 3, 37 refused the interview, 61 were not possible to contact, 12 did not show up for the interview, 9 could not be interviewed due to language barriers, in 6 the interview was incomplete, and for 9 there was no information available on the reason.

**Type of school provision**

Mainstream

**Sample demographic categories collected (including both those who did and did not participate in the study)**

None

**Participant demographic categories collected (including just those who did participate in the study)**

- Maternal age
- Maternal education
- Maternal ethnicity
- Child age
- Child sex

**Did the study use a pre-determined definition of an FASD case?**

Yes

**Guideline used for FASD case definition**

Other: IOM definition of FASD

**Measurements used: Dysmorphology**

Clinical dysmorphology assessment

**Measurements used: Parent based tools (validation status)**

- Vineland Adaptive Behaviour Scales II - VABS-II (validated)

**Measurements used: Child based tools (validation status)**

- Beery-Buktenica Developmental Test of Visual-Motor Integration
- DAS II

**Measurements used: Genetic analysis**

None

**Measurements used: Teacher based tools (validation status)**

- Vineland Adaptive Behaviour Scales II - VABS-II (validated)

**Children identified as cases seen by a clinician?**

Yes

**Prenatal alcohol exposure data measured**

- Self-reported maternal history

**Prenatal drug exposure data measured**

None

**People individual child results shared with**

Not reported

**Estimated FASD rates**

Probable:

4.80% 95% CI (3.9%, 5.7%)

**Quality Assessment (QUANDAS-2)**

Patient selection

Was a consecutive or random sample of patients enrolled? Yes

Was a case-control design avoided? Yes

Did the study avoid inappropriate exclusions? Yes

Overall - Could the selection of patient have introduced bias? Level of risk: Low

Applicability - Is there concern that the included patients do not match the review question? Level of risk: Low

Index test(s)

Were the index test results interpreted without knowledge of the results of the reference standard? No

If a threshold was used, was it pre-specified? Yes

Overall - Could the conduct or interpretation of the index test have introduced bias? Level of risk: Low

Applicability - Is there concern that the index test, its conduct, or interpretation differ from the review question? Level of risk: Low

Reference standard

Is the reference standard likely to correctly classify the target condition? Unclear - No agreed diagnostic process for FASD

Were the reference standard results interpreted without knowledge of the results of the index test? No - Reference standard includes index tests

Overall - Could the reference standard, its conduct, or its interpretation have introduced bias? Level of risk: Unclear - No agreed diagnostic process for FASD

Applicability - Is there concern that the target condition as defined by the reference standard does not match the review question? Level of risk: Unclear - No agreed diagnostic process for FASD

Flow and timing

Was there an appropriate interval between index test(s) and reference standard? Yes

Did all patients receive a reference standard? No - Only those suspected at earlier stages, plus a random sample of other children

Did patients receive the same reference standard? Yes

Were all patients included in the analysis? Yes

Overall - Could the patient flow have introduced bias? Level of risk: Low - Although not all children received a reference standard, giving one to a random subsample mitigates any risks of bias

# 10. May 2016a

May, Philip A; de Vries, Marlene M; Marais, Anna-Susan; Kalberg, Wendy O; Adnams, Colleen M; Hasken, Julie M; Tabachnick, Barbara; Robinson, Luther K; Manning, Melanie A; Jones, Kenneth Lyons; Hoyme, Derek; Seedat, Soraya; Parry, Charles D H; Hoyme, H Eugene. The continuum of fetal alcohol spectrum disorders in four rural communities in South Africa: Prevalence and characteristics. Drug and alcohol dependence 2016;159():207-218

**Study design**

Active case ascertainment

**Country study conducted in**

South Africa

**Was there cocreation of the design of the research study by people with lived experience of FASD?**

Not reported

**Number of stages in screening design**

3 stages

**Consent process**

Opt in

**Pre-screening variables**

- Height
- Weight
- Head circumference

**Study exclusion criteria**

None reported

**Participation rates**

Stage 1: 72.6% (1354/1866)

Stage 2: 97.8% (1064/1088)

Stage 3: 99.1% (738/745)

**Non-participation data**

Stage 1 reasons: Lack of consent

Stage 2 reasons: Absent on clinic day or follow up days

Stage 2 reason: Not able to interview

**Type of school provision**

Mainstream

**Sample demographic categories collected (including both those who did and did not participate in the study)**

None

**Participant demographic categories collected (including just those who did participate in the study)**

- Maternal age
- Maternal education
- Maternal ethnicity
- Household income
- Child age
- Child sex

**Did the study use a pre-determined definition of an FASD case?**

Yes

**Guideline used for FASD case definition**

Other: Hoyme et al.

**Measurements used: Dysmorphology**

Clinical dysmorphology assessment

**Measurements used: Parent based tools (validation status)**

None

**Measurements used: Child based tools (validation status)**

- WISC V (validated)
- Tests of Reception Grammar
- Ravens Coloured Progressive Matrices

**Measurements used: Genetic analysis**

None

**Measurements used: Teacher based tools (validation status)**

None

**Children identified as cases seen by a clinician?**

No

**Prenatal alcohol exposure data measured**

- Maternal Risk Questionnaire

**Prenatal drug exposure data measured**

Not reported

**People individual child results shared with**

Not reported

**Estimated FASD rates**

Probable FASD: 18.3%

Possible FASD: 25.8% 95% CI (23.8-31.6)

**Quality Assessment (QUANDAS-2)**

Patient selection

Was a consecutive or random sample of patients enrolled? Yes

Was a case-control design avoided? Yes

Did the study avoid inappropriate exclusions? Yes

Overall - Could the selection of patient have introduced bias? Level of risk: Low

Applicability - Is there concern that the included patients do not match the review question? Level of risk: Low

Index test(s)

Were the index test results interpreted without knowledge of the results of the reference standard? Yes

If a threshold was used, was it pre-specified? Yes

Overall - Could the conduct or interpretation of the index test have introduced bias? Level of risk: Low

Applicability - Is there concern that the index test, its conduct, or interpretation differ from the review question? Level of risk: Low

Reference standard

Is the reference standard likely to correctly classify the target condition? Yes

Were the reference standard results interpreted without knowledge of the results of the index test? Yes

Overall - Could the reference standard, its conduct, or its interpretation have introduced bias? Level of risk: Low

Applicability - Is there concern that the target condition as defined by the reference standard does not match the review question? Level of risk: Low

Flow and timing

Was there an appropriate interval between index test(s) and reference standard? Yes

Did all patients receive a reference standard? No - Only those suspected at earlier stages, plus a random sample of other children

Did patients receive the same reference standard? Yes

Were all patients included in the analysis? Yes

Overall - Could the patient flow have introduced bias? Level of risk: Low - Although not all children received a reference standard, giving one to a random subsample mitigates any risks of bias

# 11. May 2016b

May, Philip A; Marais, Anna-Susan; de Vries, Marlene M; Kalberg, Wendy O; Buckley, David; Hasken, Julie M; Adnams, Colleen M; Barnard, Ronel; Joubert, Belinda; Cloete, Marise; Tabachnick, Barbara; Robinson, Luther K; Manning, Melanie A; Jones, Kenneth Lyons; Bezuidenhout, Heidre; Seedat, Soraya; Parry, Charles D H; Hoyme, H Eugene. The continuum of fetal alcohol spectrum disorders in a community in South Africa: Prevalence and characteristics in a fifth sample. Drug and alcohol dependence 2016;168():274-286

**Study design**

Active case ascertainment

**Country study conducted in**

South Africa

**Was there cocreation of the design of the research study by people with lived experience of FASD?**

Not reported

**Number of stages in screening design**

3 stages

**Consent process**

Opt in

**Pre-screening variables**

- Height
- Weight
- Head circumference

**Study exclusion criteria**

None reported

**Participation rates**

Stage 1: 73.5% (862/1172)

Stage 2: 99.3% (729/734)

Stage 3: 94.8% (532/561)

**Non-participation data**

Stage 1 reasons: Lack of consent

Stage 2 reasons: Absent on clinic day or follow up days

Stage 2 reason: Not able to interview or moved out of area

**Type of school provision**

Mainstream

**Sample demographic categories collected (including both those who did and did not participate in the study)**

None

**Participant demographic categories collected (including just those who did participate in the study)**

- Maternal age
- Maternal education
- Maternal ethnicity
- Household income
- Child age
- Child sex

**Did the study use a pre-determined definition of an FASD case?**

Yes

**Guideline used for FASD case definition**

Other: Hoyme 2016

**Measurements used: Dysmorphology**

Not reported

**Measurements used: Parent based tools (validation status)**

- Personal Behaviours Checklist

**Measurements used: Child based tools (validation status)**

- WISC V (validated)
- Tests of Reception Grammar
- Ravens Coloured Progressive Matrices

**Measurements used: Genetic analysis**

None

**Measurements used: Teacher based tools (validation status)**

- Teacher Report form

**Children identified as cases seen by a clinician?**

No

**Prenatal alcohol exposure data measured**

- Maternal Risk Questionnaire

**Prenatal drug exposure data measured**

- Illicit drugs
- Tobacco

**People individual child results shared with**

Not reported

**Estimated FASD rates**

Probable: 17.0%-23.0%

**Quality Assessment (QUANDAS-2)**

Patient selection

Was a consecutive or random sample of patients enrolled? Yes

Was a case-control design avoided? Yes

Did the study avoid inappropriate exclusions? Yes

Overall - Could the selection of patient have introduced bias? Level of risk: Low

Applicability - Is there concern that the included patients do not match the review question? Level of risk: Low

Index test(s)

Were the index test results interpreted without knowledge of the results of the reference standard? Yes

If a threshold was used, was it pre-specified? Yes

Overall - Could the conduct or interpretation of the index test have introduced bias? Level of risk: Low

Applicability - Is there concern that the index test, its conduct, or interpretation differ from the review question? Level of risk: Low

Reference standard

Is the reference standard likely to correctly classify the target condition? Yes

Were the reference standard results interpreted without knowledge of the results of the index test? Unclear

Overall - Could the reference standard, its conduct, or its interpretation have introduced bias? Level of risk: Low

Applicability - Is there concern that the target condition as defined by the reference standard does not match the review question? Level of risk: Low

Flow and timing

Was there an appropriate interval between index test(s) and reference standard? Yes

Did all patients receive a reference standard? No - Only those suspected at earlier stages, plus a random sample of other children

Did patients receive the same reference standard? Yes

Were all patients included in the analysis? Yes

Overall - Could the patient flow have introduced bias? Level of risk: Low - Although not all children received a reference standard, giving one to a random subsample mitigates any risks of bias

# 12. May 2017

May, Philip A; De Vries, Marlene M; Marais, Anna-Susan; Kalberg, Wendy O; Buckley, David; Adnams, Colleen M; Hasken, Julie M; Tabachnick, Barbara; Robinson, Luther K; Manning, Melanie A; Bezuidenhout, Heidre; Adam, Margaret P; Jones, Kenneth L; Seedat, Soraya; Parry, Charles D H; Hoyme, H Eugene. Replication of High Fetal Alcohol Spectrum Disorders Prevalence Rates, Child Characteristics, and Maternal Risk Factors in a Second Sample of Rural Communities in South Africa. International journal of environmental research and public health 2017;14(5):

**Study design**

Active case ascertainment

**Country study conducted in**

South Africa

**Was there cocreation of the design of the research study by people with lived experience of FASD?**

Not reported

**Number of stages in screening design**

3 stages

**Consent process**

Opt in

**Pre-screening variables**

- Height
- Weight
- Head circumference

**Study exclusion criteria**

None reported

**Participation rates**

Consent for stage 1: 74.8% (1083/1448)

Participated in stage 2: 98.2% (866/882)

Participated in stage 3 (child testing): 98.6% (625/634)

Participated in stage 3 (maternal interview): 76.8% (487/634)

REM 33.6% (487/1448)

**Non-participation data**

At stage 1, all not included either refused consent or did not return consent.

At stage 2, all not included were absent on clinic or follow-up days.

No information is provided on reasons for non-participation at stage 3.

**Type of school provision**

Mainstream

**Sample demographic categories collected (including both those who did and did not participate in the study)**

None

**Participant demographic categories collected (including just those who did participate in the study)**

- Maternal age
- Maternal education
- Maternal ethnicity
- Household income
- Child age
- Child sex

**Did the study use a pre-determined definition of an FASD case?**

Yes

**Guideline used for FASD case definition**

Other: IOM diagnostic criteria

**Measurements used: Dysmorphology**

- Clinical dysmorphology assessment
- 2D imaging

**Measurements used: Parent based tools (validation status)**

None

**Measurements used: Child based tools (validation status)**

- Tests of Reception Grammar
- Ravens Coloured Progressive Matrices
- Digit Span (subtest) of the Wechsler Intelligence Scales for Children

**Measurements used: Genetic analysis**

None

**Measurements used: Teacher based tools (validation status)**

- Teacher Report form

**Children identified as cases seen by a clinician?**

No

**Prenatal alcohol exposure data measured**

- Self-reported maternal history

**Prenatal drug exposure data measured**

- Illicit drugs
- Tobacco

**People individual child results shared with**

Not reported

**Estimated FASD rates**

Probable FASD

27.6% (25.3%, 29.9%)

**Quality Assessment (QUANDAS-2)**

Patient selection

Was a consecutive or random sample of patients enrolled? Yes

Was a case-control design avoided? Yes

Did the study avoid inappropriate exclusions? Yes

Overall - Could the selection of patient have introduced bias? Level of risk: Low

Applicability - Is there concern that the included patients do not match the review question? Level of risk: Low

Index test(s)

Were the index test results interpreted without knowledge of the results of the reference standard? Yes

If a threshold was used, was it pre-specified? Yes

Overall - Could the conduct or interpretation of the index test have introduced bias? Level of risk: Low

Applicability - Is there concern that the index test, its conduct, or interpretation differ from the review question? Level of risk: Low

Reference standard

Is the reference standard likely to correctly classify the target condition? Unclear - No agreed standard for FASD diagnosis

Were the reference standard results interpreted without knowledge of the results of the index test? No - Reference standard includes index tests

Overall - Could the reference standard, its conduct, or its interpretation have introduced bias? Level of risk: Unclear - No agreed standard for FASD diagnosis

Applicability - Is there concern that the target condition as defined by the reference standard does not match the review question? Level of risk: Unclear - No agreed standard for FASD diagnosis

Flow and timing

Was there an appropriate interval between index test(s) and reference standard? Yes

Did all patients receive a reference standard? No

Did patients receive the same reference standard? Yes

Were all patients included in the analysis? Yes

Overall - Could the patient flow have introduced bias? Level of risk: Low - Although not all children given a reference standard, giving one to a random subsample should mitigate the risk of bias.

# 13. May 2000

May, P A; Brooke, L; Gossage, J P; Croxford, J; Adnams, C; Jones, K L; Robinson, L; Viljoen, D. Epidemiology of fetal alcohol syndrome in a South African community in the Western Cape Province. American journal of public health. 2000;90(12):1905-1912

**Study design**

Active case ascertainment

**Country study conducted in**

South Africa

**Was there cocreation of the design of the research study by people with lived experience of FASD?**

No co-creation, some stakeholder involvement

**Number of stages in screening design**

2 stages

**Consent process**

- Opt out at stage 1
- Opt in at stage 2

**Pre-screening variables**

- Height
- Weight
- Head circumference

**Study exclusion criteria**

None reported

**Participation rates**

92.5% at school level 100% at stage 1

**Non-participation data**

None reported

**Type of school provision**

- Mainstream
- Special educational needs
- Other: out of school children audited not assessed

**Sample demographic categories collected (including both those who did and did not participate in the study)**

- Maternal education
- Maternal ethnicity
- Child age
- Child sex

**Participant demographic categories collected (including just those who did participate in the study)**

- Maternal age
- Maternal ethnicity
- Household income
- Child age
- Child sex

**Did the study use a pre-determined definition of an FASD case?**

Yes

**Guideline used for FASD case definition**

Hoyme et al

**Measurements used: Dysmorphology**

Clinical dysmorphology assessment

**Measurements used: Parent based tools (validation status)**

Not reported

**Measurements used: Child based tools (validation status)**

- Griffiths Mental Development Scale

**Measurements used: Genetic analysis**

None

**Measurements used: Teacher based tools (validation status)**

Not reported

**Children identified as cases seen by a clinician?**

Not reported

**Prenatal alcohol exposure data measured**

- Maternal Risk Questionnaire

**Prenatal drug exposure data measured**

- Illicit drugs
- Tobacco

**People individual child results shared with**

Not reported

**Estimated FASD rates**

FAS: 4.6%

**Quality Assessment (QUANDAS-2)**

Patient selection

Was a consecutive or random sample of patients enrolled? Yes

Was a case-control design avoided? Yes

Did the study avoid inappropriate exclusions? Yes

Overall - Could the selection of patient have introduced bias? Level of risk: Low

Applicability - Is there concern that the included patients do not match the review question? Level of risk: Low

Index test(s)

Were the index test results interpreted without knowledge of the results of the reference standard? Yes

If a threshold was used, was it pre-specified? Yes

Overall - Could the conduct or interpretation of the index test have introduced bias? Level of risk: Low

Applicability - Is there concern that the index test, its conduct, or interpretation differ from the review question? Level of risk: Low

Reference standard

Is the reference standard likely to correctly classify the target condition? Yes – the study only looked for full FAS

Were the reference standard results interpreted without knowledge of the results of the index test? Yes

Overall - Could the reference standard, its conduct, or its interpretation have introduced bias? Level of risk: Low

Applicability - Is there concern that the target condition as defined by the reference standard does not match the review question? Level of risk: High – the study only looked for full FAS

Flow and timing

Was there an appropriate interval between index test(s) and reference standard? Yes

Did all patients receive a reference standard? No - Only those suspected at earlier stages, plus a random sample of other children

Did patients receive the same reference standard? Yes

Were all patients included in the analysis? Yes

Overall - Could the patient flow have introduced bias? Level of risk: Low - Although not all children received a reference standard, giving one to a random subsample mitigates any risks of bias

# 14. May 2020a

May, Philip A; Hasken, Julie M; Stegall, Julie M; Mastro, Heather A; Kalberg, Wendy O; Buckley, David; Brooks, Marita; Hedrick, Dixie M; Ortega, Marian A; Elliott, Amy J; Tabachnick, Barbara G; Abdul-Rahman, Omar; Adam, Margaret P; Robinson, Luther K; Manning, Melanie A; Jewett, Tamison; Hoyme, H Eugene. Fetal Alcohol Spectrum Disorders in a Southeastern County of the United States: Child Characteristics and Maternal Risk Traits. Alcoholism, clinical and experimental research 2020;44(4):939-959

**Study design**

Active case ascertainment

**Country study conducted in**

USA

**Was there cocreation of the design of the research study by people with lived experience of FASD?**

Not reported

**Number of stages in screening design**

3 stages

**Consent process**

Opt in

**Pre-screening variables**

- Height
- Weight
- Head circumference
- School opts child in
- Parent opts child in
- Twin of participating child

**Study exclusion criteria**

None reported

**Participation rates**

Consent for stage 1: 49.8% (1439/2887)

Participation in stage 2: 96.5% (825/855)

Consent for stage 3 (child assessment): 78.2% (469/600)

Consent for stage 3 (maternal interview): 63.3% (380/600)

**Non-participation data**

Of the 1,448 not consenting at stage 1, 778 refused consent and 670 did not return the consent form.

Of the 30 not participating at stage 2, 11 dropped out of the study, 14 had moved out of the area, and the random were absent when the tests took place.

Of the 131 not consenting to child assessment at stage 3, 5 refused consent, 51 were unable to schedule an assessment, 13 did not attend a scheduled assessment, 39 had moved out of the area and 23 dropped out of the study.

Of the 131 not consenting to maternal interviews at stage 3, 21 refused consent, 103 were unable to schedule an interview, 30 did not attend a scheduled interview, 38 had moved out of the area and 28 dropped out of the study.

**Type of school provision**

Mainstream

**Sample demographic categories collected (including both those who did and did not participate in the study)**

None

**Participant demographic categories collected (including just those who did participate in the study)**

- Maternal age
- Maternal education
- Maternal ethnicity
- Household income
- Child age
- Child sex

**Did the study use a pre-determined definition of an FASD case?**

Yes

**Guideline used for FASD case definition**

Revised IOM diagnostic guidelines for FASD (Hoyme et al., 2005) were used and consensus cut-off values were established by the investigators and CoFASP advisory group (Hoyme et al., 2016).

**Measurements used: Dysmorphology**

Clinical dysmorphology assessment

**Measurements used: Parent based tools (validation status)**

- Child Behaviour Checklist

**Measurements used: Child based tools (validation status)**

- NEPSY-II (validated)
- BRACKEN
- DAS II
- Developmental Test of Visual-Motor Integration (VMI)

**Measurements used: Genetic analysis**

None

**Measurements used: Teacher based tools (validation status)**

- Teacher Report form
- Vineland Adaptive Behaviour Scales II - VABS-II (validated)

**Children identified as cases seen by a clinician?**

No

**Prenatal alcohol exposure data measured**

- Self-reported maternal history

**Prenatal drug exposure data measured**

- Illicit drugs
- Other prescription drugs
- Tobacco

**People individual child results shared with**

Not reported

**Estimated FASD rates**

4.90% Not reported

**Quality Assessment (QUANDAS-2)**

Patient selection

Was a consecutive or random sample of patients enrolled? Yes

Was a case-control design avoided? Yes

Did the study avoid inappropriate exclusions? Yes

Overall - Could the selection of patient have introduced bias? Level of risk: Low

Applicability - Is there concern that the included patients do not match the review question? Level of risk: Low

Index test(s)

Were the index test results interpreted without knowledge of the results of the reference standard? Yes

If a threshold was used, was it pre-specified? Yes

Overall - Could the conduct or interpretation of the index test have introduced bias? Level of risk: Low

Applicability - Is there concern that the index test, its conduct, or interpretation differ from the review question? Level of risk: Low

Reference standard

Is the reference standard likely to correctly classify the target condition? Unclear - No agreed diagnostic criteria for FASD

Were the reference standard results interpreted without knowledge of the results of the index test? No - Index tests form part of the reference standard

Overall - Could the reference standard, its conduct, or its interpretation have introduced bias? Level of risk: Unclear - No agreed diagnostic criteria for FASD

Applicability - Is there concern that the target condition as defined by the reference standard does not match the review question? Level of risk: Unclear - No agreed diagnostic criteria for FASD

Flow and timing

Was there an appropriate interval between index test(s) and reference standard? Yes

Did all patients receive a reference standard? No - Only those suspected of FASD, plus a random control subsample

Did patients receive the same reference standard? Yes

Were all patients included in the analysis? Yes

Overall - Could the patient flow have introduced bias? Level of risk: Low - Although not all participants given the references standard, the inclusion of a random subsample of those not suspected of FASD should mitigate the risk of bias.

# 15. May 2020b

May, Philip A; Hasken, Julie M; Baete, Amy; Russo, Jaymi; Elliott, Amy J; Kalberg, Wendy O; Buckley, David; Brooks, Marita; Ortega, Marian A; Hedrick, Dixie M; Tabachnick, Barbara G; Abdul-Rahman, Omar; Adam, Margaret P; Jewett, Tamison; Robinson, Luther K; Manning, Melanie A; Hoyme, H Eugene. Fetal Alcohol Spectrum Disorders in a Midwestern City: Child Characteristics, Maternal Risk Traits, and Prevalence. Alcoholism, clinical and experimental research 2020;44(4):919-938

**Study design**

Active case ascertainment

**Country study conducted in**

USA

**Was there cocreation of the design of the research study by people with lived experience of FASD?**

Not reported

**Number of stages in screening design**

3 stages

**Consent process**

Opt in

**Pre-screening variables**

- Height
- Weight
- Head circumference
- School opts child in
- Parent opts child in

**Study exclusion criteria**

None reported

**Participation rates**

Stage 1: 53.6% (380/709)

Stage 2: 99.7% (379/380)

Stage 3: 62.3% (236/379)

**Non-participation data**

Stage 1 reason: Lack of consent

Stage 2 reason: Absent for clinic

Stage 2 reasons: Unable to schedule, refused, no show

**Type of school provision**

Mainstream

**Sample demographic categories collected (including both those who did and did not participate in the study)**

None

**Participant demographic categories collected (including just those who did participate in the study)**

- Maternal age
- Maternal education
- Maternal ethnicity
- Household income
- Child age
- Child sex

**Did the study use a pre-determined definition of an FASD case?**

Yes

**Guideline used for FASD case definition**

Other: Hoyme 2016

**Measurements used: Dysmorphology**

Clinical dysmorphology assessment

**Measurements used: Parent based tools (validation status)**

- Vineland Adaptive Behaviour Scales II - VABS-II (validated)
- Achenback
- Child Behaviour Checklist

**Measurements used: Child based tools (validation status)**

- NEPSY (validated)
- BRACKEN
- Beery-Buktenica Developmental Test of Visual-Motor Integration
- DAS II

**Measurements used: Genetic analysis**

Not reported

**Measurements used: Teacher based tools (validation status)**

- Teacher Report form

**Children identified as cases seen by a clinician?**

Yes

**Prenatal alcohol exposure data measured**

- Maternal Risk Questionnaire

**Prenatal drug exposure data measured**

- Illicit drugs
- Other non-prescription drugs
- Tobacco

**People individual child results shared with**

Not reported

**Estimated FASD rates**

1.4% - 4.1%

**Quality Assessment (QUANDAS-2)**

Patient selection

Was a consecutive or random sample of patients enrolled? Yes

Was a case-control design avoided? Yes

Did the study avoid inappropriate exclusions? Yes

Overall - Could the selection of patient have introduced bias? Level of risk: Low

Applicability - Is there concern that the included patients do not match the review question? Level of risk: Low

Index test(s)

Were the index test results interpreted without knowledge of the results of the reference standard? Yes

If a threshold was used, was it pre-specified? Yes

Overall - Could the conduct or interpretation of the index test have introduced bias? Level of risk: Low

Applicability - Is there concern that the index test, its conduct, or interpretation differ from the review question? Level of risk: Low

Reference standard

Is the reference standard likely to correctly classify the target condition? Yes

Were the reference standard results interpreted without knowledge of the results of the index test? Unclear

Overall - Could the reference standard, its conduct, or its interpretation have introduced bias? Level of risk: Low

Applicability - Is there concern that the target condition as defined by the reference standard does not match the review question? Level of risk: Low

Flow and timing

Was there an appropriate interval between index test(s) and reference standard? Yes

Did all patients receive a reference standard? No - Only those suspected at earlier stages, plus a random sample of other children

Did patients receive the same reference standard? Yes

Were all patients included in the analysis? Yes

Overall - Could the patient flow have introduced bias? Level of risk: Low - Although not all children received a reference standard, giving one to a random subsample mitigates any risks of bias

# 16. May 2020c

May, Philip A; Hasken, Julie M; Bozeman, Rosemary; Jones, Jo-Viviane; Burns, Mary Kay; Goodover, Joelene; Kalberg, Wendy O; Buckley, David; Brooks, Marita; Ortega, Marian A; Elliott, Amy J; Hedrick, Dixie M; Tabachnick, Barbara G; Abdul-Rahman, Omar; Adam, Margaret P; Jewett, Tamison; Robinson, Luther K; Manning, Melanie A; Hoyme, H Eugene. Fetal Alcohol Spectrum Disorders in a Rocky Mountain Region City: Child Characteristics, Maternal Risk Traits, and Prevalence. Alcoholism, clinical and experimental research 2020;44(4):900-918

**Study design**

Active case ascertainment

**Country study conducted in**

USA

**Was there cocreation of the design of the research study by people with lived experience of FASD?**

Not reported

**Number of stages in screening design**

3 stages

**Consent process**

Opt in

**Pre-screening variables**

- Height
- Weight
- Head circumference
- School opts child in
- Parent opts child in
- Twin of participating child

**Study exclusion criteria**

None reported

**Participation rates**

Stage 1: 62.4% (571/915)

Stage 2: 82.6% (265/321)

Stage 3: 99.4% (167/168)

**Non-participation data**

Stage 1 reason: Lack of consent

Stage 2 reason: Absent for exam

Stage 2 reason: Moved

**Type of school provision**

Mainstream

**Sample demographic categories collected (including both those who did and did not participate in the study)**

- Ethnicity
- Education
- Religion

**Participant demographic categories collected (including just those who did participate in the study)**

- Maternal age
- Maternal education

**Did the study use a pre-determined definition of an FASD case?**

Yes

**Guideline used for FASD case definition**

Revised Institute of Medicine (IOM) diagnostic guidelines for FASD (Hoyme et al., 2005) were used along with revised cut-off values established by the CoFASP advisory group (Hoyme et al., 2016)

**Measurements used: Dysmorphology**

- Clinical dysmorphology assessment
- 2D imaging

**Measurements used: Parent based tools (validation status)**

- Child Behavior Checklist
- Vineland Adaptive Behavior Scales

**Measurements used: Child based tools (validation status)**

- DAS II
- NEPSY-II
- Developmental Test of Visual-Motor Integration
- Bracken Basic Concept Scale

**Measurements used: Genetic analysis**

None

**Measurements used: Teacher based tools (validation status)**

- Child Behavior Checklist
- Vineland Adaptive Behavior Scales

**Children identified as cases seen by a clinician?**

Not reported

**Prenatal alcohol exposure data measured**

- Self-reported maternal history

**Prenatal drug exposure data measured**

- Tobacco use
- Drugs in pregnancy
- Marijuana use
- Club drug use

**People individual child results shared with**

Not reported

**Estimated FASD rates**

FAS: 0.3-0.6%

FASD: 3.5-8.3%

**Quality Assessment (QUANDAS-2)**

Patient selection

Was a consecutive or random sample of patients enrolled? Yes

Was a case-control design avoided? Yes

Did the study avoid inappropriate exclusions? Yes

Overall - Could the selection of patient have introduced bias? Level of risk: Low

Applicability - Is there concern that the included patients do not match the review question? Level of risk: Low

Index test(s)

Were the index test results interpreted without knowledge of the results of the reference standard? Yes

If a threshold was used, was it pre-specified? Yes

Overall - Could the conduct or interpretation of the index test have introduced bias? Level of risk: Low

Applicability - Is there concern that the index test, its conduct, or interpretation differ from the review question? Level of risk: Low

Reference standard

Is the reference standard likely to correctly classify the target condition? Unclear - No agreed diagnostic criteria for FASD

Were the reference standard results interpreted without knowledge of the results of the index test? No - Index tests form part of the reference standard

Overall - Could the reference standard, its conduct, or its interpretation have introduced bias? Level of risk: Unclear - No agreed diagnostic criteria for FASD

Applicability - Is there concern that the target condition as defined by the reference standard does not match the review question? Level of risk: Unclear - No agreed diagnostic criteria for FASD

Flow and timing

Was there an appropriate interval between index test(s) and reference standard? Yes

Did all patients receive a reference standard? No - Only those suspected at earlier stages, plus a random sample of other children

Did patients receive the same reference standard? Yes

Were all patients included in the analysis? Yes

Overall - Could the patient flow have introduced bias? Level of risk: Low - Although not all children received a reference standard, giving one to a random subsample mitigates any risks of bias

# 17. May 2021a

May, Philip A; Marais, Anna-Susan; De Vries, Marlene M; Buckley, David; Kalberg, Wendy O; Hasken, Julie M; Stegall, Julie M; Hedrick, Dixie M; Robinson, Luther K; Manning, Melanie A; Tabachnick, Barbara G; Seedat, Soraya; Parry, Charles D H; Hoyme, H Eugene. The prevalence, child characteristics, and maternal risk factors for the continuum of fetal alcohol spectrum disorders: A sixth population-based study in the same South African community. Drug and alcohol dependence 2021;218():108408

**Study design**

Active case ascertainment

**Country study conducted in**

South Africa

**Was there cocreation of the design of the research study by people with lived experience of FASD?**

Not reported

**Number of stages in screening design**

3 stages

**Consent process**

Opt in

**Pre-screening variables**

- Height
- Weight
- Head circumference

**Study exclusion criteria**

None reported

**Participation rates**

Stage 1: 65.1% (735/1128)

Stage 2: 98.8% (554/561)

Stage 3: 92.4% (364/394)

**Non-participation data**

Stage 1 reason: Lack of consent

Stage 2 reasons: Absent on clinic day or follow-up days

Stage 2 reason: Not able to interview

**Type of school provision**

Mainstream

**Sample demographic categories collected (including both those who did and did not participate in the study)**

None

**Participant demographic categories collected (including just those who did participate in the study)**

- Maternal age
- Maternal education
- Maternal ethnicity
- Household income
- Child age
- Child sex

**Did the study use a pre-determined definition of an FASD case?**

Yes

**Guideline used for FASD case definition**

Other: Hoyme 2016

**Measurements used: Dysmorphology**

Clinical dysmorphology assessment

**Measurements used: Parent based tools (validation status)**

None

**Measurements used: Child based tools (validation status)**

- WISC V (validated)
- Tests of Reception Grammar
- Ravens Coloured Progressive Matrices

**Measurements used: Genetic analysis**

None

**Measurements used: Teacher based tools (validation status)**

None

**Children identified as cases seen by a clinician?**

No

**Prenatal alcohol exposure data measured**

- Maternal Risk Questionnaire

**Prenatal drug exposure data measured**

- Illicit drugs
- Other prescription drugs
- Other non-prescription drugs
- Tobacco

**People individual child results shared with**

Not reported

**Estimated FASD rates**

Probable FASD: 30.6% (95% CI: 27.9%-33.3%)

**Quality Assessment (QUANDAS-2)**

Patient selection

Was a consecutive or random sample of patients enrolled? Yes

Was a case-control design avoided? Yes

Did the study avoid inappropriate exclusions? Yes

Overall - Could the selection of patient have introduced bias? Level of risk: Low

Applicability - Is there concern that the included patients do not match the review question? Level of risk: Low

Index test(s)

Were the index test results interpreted without knowledge of the results of the reference standard? Yes

If a threshold was used, was it pre-specified? Yes

Overall - Could the conduct or interpretation of the index test have introduced bias? Level of risk: Low

Applicability - Is there concern that the index test, its conduct, or interpretation differ from the review question? Level of risk: Low

Reference standard

Is the reference standard likely to correctly classify the target condition? Yes

Were the reference standard results interpreted without knowledge of the results of the index test? Yes

Overall - Could the reference standard, its conduct, or its interpretation have introduced bias? Level of risk: Low

Applicability - Is there concern that the target condition as defined by the reference standard does not match the review question? Level of risk: Low

Flow and timing

Was there an appropriate interval between index test(s) and reference standard? Yes

Did all patients receive a reference standard? No - Only those suspected at earlier stages, plus a random sample of other children

Did patients receive the same reference standard? Yes

Were all patients included in the analysis? Yes

Overall - Could the patient flow have introduced bias? Level of risk: Low - Although not all children received a reference standard, giving one to a random subsample mitigates any risks of bias

# 18. May 2021b

May, Philip A; Hasken, Julie M; Hooper, Stephen R; Hedrick, Dixie M; Jackson-Newsom, Julia; Mullis, Chalise E; Dobyns, Elizabeth; Kalberg, Wendy O; Buckley, David; Robinson, Luther K; Abdul-Rahman, Omar; Adam, Margaret P; Manning, Melanie A; Jewett, Tamison; Hoyme, H Eugene. Estimating the community prevalence, child traits, and maternal risk factors of fetal alcohol spectrum disorders (FASD) from a random sample of school children. Drug and alcohol dependence 2021;227:108918

**Study design**

Other: Simple random sample

**Country study conducted in**

USA

**Was there cocreation of the design of the research study by people with lived experience of FASD?**

Not reported

**Number of stages in screening design**

2 stages

**Consent process**

Opt in

**Pre-screening variables**

None used

**Study exclusion criteria**

None reported

**Participation rates**

Stage 1: 32.0%: (231/721)

Stage 2: 85.7% (72/84)

**Non-participation data**

Stage 1: Lack of consent

Stage 2: Not reported

**Type of school provision**

- Mainstream
- Special educational needs

**Sample demographic categories collected (including both those who did and did not participate in the study)**

None

**Participant demographic categories collected (including just those who did participate in the study)**

- Maternal age
- Maternal education
- Maternal ethnicity
- Household income
- Child age
- Child sex

**Did the study use a pre-determined definition of an FASD case?**

Yes

**Guideline used for FASD case definition**

Other: Hoyme 2016

**Measurements used: Dysmorphology**

Clinical dysmorphology assessment

**Measurements used: Parent based tools (validation status)**

- Vineland Adaptive Behaviour Scales II - VABS-II (validated)
- Child Behaviour Checklist

**Measurements used: Child based tools (validation status)**

- NEPSY (validated)
- BRACKEN
- Beery-Buktenica Developmental Test of Visual-Motor Integration
- DAS II

**Measurements used: Genetic analysis**

Not reported

**Measurements used: Teacher based tools (validation status)**

- Teacher Report form

**Children identified as cases seen by a clinician?**

No

**Prenatal alcohol exposure data measured**

- Maternal Risk Questionnaire

**Prenatal drug exposure data measured**

- Illicit drugs
- Other prescription drugs
- Tobacco

**People individual child results shared with**

Not reported

**Estimated FASD rates**

Probable FASD: 7.14% 1.64 -12.65%

**Quality Assessment (QUANDAS-2)**

Patient selection

Was a consecutive or random sample of patients enrolled? Yes

Was a case-control design avoided? Yes

Did the study avoid inappropriate exclusions? Yes

Overall - Could the selection of patient have introduced bias? Level of risk: Low

Applicability - Is there concern that the included patients do not match the review question? Level of risk: Low

Index test(s)

Were the index test results interpreted without knowledge of the results of the reference standard? Yes

If a threshold was used, was it pre-specified? Yes

Overall - Could the conduct or interpretation of the index test have introduced bias? Level of risk: Low

Applicability - Is there concern that the index test, its conduct, or interpretation differ from the review question? Level of risk: Low

Reference standard

Is the reference standard likely to correctly classify the target condition? Yes

Were the reference standard results interpreted without knowledge of the results of the index test? Yes

Overall - Could the reference standard, its conduct, or its interpretation have introduced bias? Level of risk: Low

Applicability - Is there concern that the target condition as defined by the reference standard does not match the review question? Level of risk: Low

Flow and timing

Was there an appropriate interval between index test(s) and reference standard? Yes

Did all patients receive a reference standard? No – Study design

Did patients receive the same reference standard? No

Were all patients included in the analysis? No

Overall - Could the patient flow have introduced bias? Level of risk: High - All participants not consenting to be in the study were assumed not to have FASD, which is likely to bias prevalence estimates

# 19. May 2022

May, Philip A; de Vries, Marlene M; Marais, Anna-Susan; Kalberg, Wendy O; Buckley, David; Hasken, Julie M; Abdul-Rahman, Omar; Robinson, Luther K; Manning, Melanie A; Seedat, Soraya; Parry, Charles D H; Hoyme, H Eugene. The prevalence of fetal alcohol spectrum disorders in rural communities in South Africa: A third regional sample of child characteristics and maternal risk factors. Alcoholism, clinical and experimental research 2022;46(10):1819-1836

**Study design**

Active case ascertainment

**Country study conducted in**

South Africa

**Was there cocreation of the design of the research study by people with lived experience of FASD?**

Not reported

**Number of stages in screening design**

3 stages

**Consent process**

Opt in

**Pre-screening variables**

- Height
- Weight
- Head circumference

**Study exclusion criteria**

None reported

**Participation rates**

Consent for stage 1: 76.6% (888/1158)

Participation in stage 2: 98.8% (687/695)

Consent at stage 3 (child assessment): 98.8% (554/561)

Consent at stage 3 (maternal interview): 97.5% (547/561)

**Non-participation data**

All excluded at stage 1 either refused consent or did not return the consent form.

All not participating at stage 2 were absent on clinic or follow-up days.

**Type of school provision**

Mainstream

**Sample demographic categories collected (including both those who did and did not participate in the study)**

None

**Participant demographic categories collected (including just those who did participate in the study)**

- Maternal age
- Maternal education
- Household income
- Child age
- Child sex

**Did the study use a pre-determined definition of an FASD case?**

Yes

**Guideline used for FASD case definition**

Other: IOM diagnostic criteria

**Measurements used: Dysmorphology**

Clinical dysmorphology assessment

**Measurements used: Parent based tools (validation status)**

None

**Measurements used: Child based tools (validation status)**

- Kaufman Assessment Battery for Children (KABC-II)

**Measurements used: Genetic analysis**

None

**Measurements used: Teacher based tools (validation status)**

- Teacher Report form

**Children identified as cases seen by a clinician?**

No

**Prenatal alcohol exposure data measured**

- Self-reported maternal history

**Prenatal drug exposure data measured**

- Tobacco

**People individual child results shared with**

Not reported

**Estimated FASD rates**

Probable FASD: 31.00%	(28.3%, 33.7%)

**Quality Assessment (QUANDAS-2)**

Patient selection

Was a consecutive or random sample of patients enrolled? Yes

Was a case-control design avoided? Yes

Did the study avoid inappropriate exclusions? Yes

Overall - Could the selection of patient have introduced bias? Level of risk: Low

Applicability - Is there concern that the included patients do not match the review question? Level of risk: Low

Index test(s)

Were the index test results interpreted without knowledge of the results of the reference standard? Yes

If a threshold was used, was it pre-specified? Yes

Overall - Could the conduct or interpretation of the index test have introduced bias? Level of risk: Low

Applicability - Is there concern that the index test, its conduct, or interpretation differ from the review question? Level of risk: Low

Reference standard

Is the reference standard likely to correctly classify the target condition? Unclear - No standard definition for FASD

Were the reference standard results interpreted without knowledge of the results of the index test? No - Reference standard includes index tests

Overall - Could the reference standard, its conduct, or its interpretation have introduced bias? Level of risk: Unclear - No standard definition for FASD

Applicability - Is there concern that the target condition as defined by the reference standard does not match the review question? Level of risk: Unclear - No standard definition for FASD

Flow and timing

Was there an appropriate interval between index test(s) and reference standard? Yes

Did all patients receive a reference standard? No - Only those with suspected FASD, plus a random control subsample, received the reference standard

Did patients receive the same reference standard? Yes

Were all patients included in the analysis? Yes

Overall - Could the patient flow have introduced bias? Level of risk: Low - Although not all participants were given a reference standard, the use of a random subsample of participants should mitigate the risk of bias

# 20. McCarthy 2021

McCarthy, Robyn; Mukherjee, Raja A S; Fleming, Kate M; Green, Jonathan; Clayton-Smith, Jill; Price, Alan D; Allely, Clare S; Cook, Penny A. Prevalence of fetal alcohol spectrum disorder in Greater Manchester, UK: An active case ascertainment study. Alcoholism, clinical and experimental research 2021;45(11):2271-2281

**Study design**

Active case ascertainment

**Country study conducted in**

UK

**Was there cocreation of the design of the research study by people with lived experience of FASD?**

No co-creation, some stakeholder involvement

**Number of stages in screening design**

2 stages

**Consent process**

- Opt out at stage 1
- Opt in at stage 2

**Pre-screening variables**

- Height
- Weight
- Head circumference
- Child is on school's special educational needs (SEN) register
- Child is currently or has been previously under the care of the state
- School opts child in
- Parent opts child in

**Study exclusion criteria**

- Diagnosed genetic syndrome
- Postnatal brain injury
- Either parent or child unable to speak English

**Participation rates**

Stage 1: (203/220): 92%

Stage 2: (26/50): 52%

REM Stage 2: 11.8% (26/220)

**Non-participation data**

Reported as "due largely to parents not giving consent or being uncontactable.”

**Type of school provision**

Mainstream

**Sample demographic categories collected (including both those who did and did not participate in the study)**

None

**Participant demographic categories collected (including just those who did participate in the study)**

- Maternal education
- Maternal ethnicity
- Child age
- Child sex

**Did the study use a pre-determined definition of an FASD case?**

Yes

**Guideline used for FASD case definition**

Other: Case definition derived from WHO protocol for prevalence studies, and other international guidelines

**Measurements used: Dysmorphology**

2D imaging

**Measurements used: Parent based tools (validation status)**

- Vineland Adaptive Behaviour Scales II - VABS-II (validated)
- Social Communication Questionnaire - SCQ (validated)
- Communication checklist for children - CCC-2 (validated)
- Strengths and Difficulties Questionnaire - SDQ (validated)
- Short Sensory Profile - SSP (validated)

**Measurements used: Child based tools (validation status)**

- WISC V (validated)
- NEPSY-II (validated)

**Measurements used: Genetic analysis**

- Comparative genomic hybridization (CGH)

**Measurements used: Teacher based tools (validation status)**

None

**Children identified as cases seen by a clinician?**

No

**Prenatal alcohol exposure data measured**

- Maternal Risk Questionnaire

**Prenatal drug exposure data measured**

- Illicit drugs
- Anticonvulsants
- Other prescription drugs
- Other non-prescription drugs
- Tobacco

**People individual child results shared with**

- Parents/carers

**Estimated FASD rates**

Probable FASD: 1.80% (1.0%, 3.4%)

Possible FASD: 3.60% (2.1%, 6.3%)

**Quality Assessment (QUANDAS-2)**

Patient selection

Was a consecutive or random sample of patients enrolled? Yes

Was a case-control design avoided? Yes

Did the study avoid inappropriate exclusions? Yes

Overall - Could the selection of patient have introduced bias? Level of risk: Low

Applicability - Is there concern that the included patients do not match the review question? Level of risk: Low

Index test(s)

Were the index test results interpreted without knowledge of the results of the reference standard? Yes

If a threshold was used, was it pre-specified? Yes

Overall - Could the conduct or interpretation of the index test have introduced bias? Level of risk: Low

Applicability - Is there concern that the index test, its conduct, or interpretation differ from the review question? Level of risk: Low

Reference standard

Is the reference standard likely to correctly classify the target condition? Unclear - Diagnoses were made in case conferences by clinicians with expertise in the diagnosis of FASD. However, the full process for those decisions being made is somewhat unclear

Were the reference standard results interpreted without knowledge of the results of the index test? No - The index tests conducted all form part of the reference standard for an FASD diagnosis

Overall - Could the reference standard, its conduct, or its interpretation have introduced bias? Level of risk: Unclear - There is no universally agreed best practice for FASD diagnosis - therefore specific choices made for the references standard in this study may have affected the results

Applicability - Is there concern that the target condition as defined by the reference standard does not match the review question? Level of risk: Low

Flow and timing

Was there an appropriate interval between index test(s) and reference standard? Yes

Did all patients receive a reference standard? No - The reference standard was only conducted on participants who qualified for a phase 2 assessment in the study

Did patients receive the same reference standard? Unclear - As the reference standard was based on clinical judgement in case conferences, it is not possible to be certain how consistently the reference standard was applied

Were all patients included in the analysis? Yes

Overall - Could the patient flow have introduced bias? Level of risk: Unclear - Prevalence estimates from this study may be underestimates, as only people referred to stage 2 of the study received a full FASD assessment

# 21. Okulicz-Kozaryn 2017

Okulicz-Kozaryn, Katarzyna; Borkowska, Magdalena; Brzózka, Krzysztof. FASD Prevalence among Schoolchildren in Poland. Journal of applied research in intellectual disabilities 2017;30(1):61-70

**Study design**

Active case ascertainment

**Country study conducted in**

Poland

**Was there cocreation of the design of the research study by people with lived experience of FASD?**

Not reported

**Number of stages in screening design**

2 stages

**Consent process**

Opt in

**Pre-screening variables**

- Height
- Weight
- Head circumference
- School opts child in
- Parent opts child in

**Study exclusion criteria**

None reported

**Participation rates**

Consented to stage 1: 16.4% (409/2500)

Information not provided for stage 2

**Non-participation data**

No information provided other than that people did not consent

**Type of school provision**

Mainstream

**Sample demographic categories collected (including both those who did and did not participate in the study)**

None

**Participant demographic categories collected (including just those who did participate in the study)**

- Maternal age
- Maternal education
- Child age
- Child sex

**Did the study use a pre-determined definition of an FASD case?**

Yes

**Guideline used for FASD case definition**

Canadian

**Measurements used: Dysmorphology**

Clinical dysmorphology assessment

**Measurements used: Parent based tools (validation status)**

- Child Behaviour Checklist; Neurobehavioral screening tool (NST)

**Measurements used: Child based tools (validation status)**

None

**Measurements used: Genetic analysis**

None

**Measurements used: Teacher based tools (validation status)**

- Teacher Report form

**Children identified as cases seen by a clinician?**

Yes

**Prenatal alcohol exposure data measured**

- Self-reported maternal history

**Prenatal drug exposure data measured**

None

**People individual child results shared with**

Not reported

**Estimated FASD rates**

Probable: 2.0% 95% CI (1.5%, 2.6%)

**Quality Assessment (QUANDAS-2)**

Patient selection

Was a consecutive or random sample of patients enrolled? Yes

Was a case-control design avoided? Yes

Did the study avoid inappropriate exclusions? Yes

Overall - Could the selection of patient have introduced bias? Level of risk: High - Only 16% of those eligible consented to participate in the study

Applicability - Is there concern that the included patients do not match the review question? Level of risk: High - Only 16% of those eligible consented to participate in the study

Index test(s)

Were the index test results interpreted without knowledge of the results of the reference standard? Yes

If a threshold was used, was it pre-specified? Unclear - Limited information reported on interpretation of index test

Overall - Could the conduct or interpretation of the index test have introduced bias? Level of risk: Unclear - Limited information reported on interpretation of index test

Applicability - Is there concern that the index test, its conduct, or interpretation differ from the review question? Level of risk: Low

Reference standard

Is the reference standard likely to correctly classify the target condition? Unclear - No agreed standard for FASD diagnosis

Were the reference standard results interpreted without knowledge of the results of the index test? No - Index tests form part of the reference standard

Overall - Could the reference standard, its conduct, or its interpretation have introduced bias? Level of risk: Unclear - No agreed standard for FASD diagnosis

Applicability - Is there concern that the target condition as defined by the reference standard does not match the review question? Level of risk: Unclear - No agreed standard for FASD diagnosis

Flow and timing

Was there an appropriate interval between index test(s) and reference standard? Yes

Did all patients receive a reference standard? Yes

Did patients receive the same reference standard? Yes

Were all patients included in the analysis? No - All participants not consenting to be in the study were assumed not to have FASD

Overall - Could the patient flow have introduced bias? Level of risk: High - All participants not consenting to be in the study were assumed not to have FASD, which is likely to bias prevalence estimates

# 22. Olivier 2013

Olivier, L; Urban, M; Chersich, M; Temmerman, M; Viljoen, D. Burden of fetal alcohol syndrome in a rural West Coast area of South Africa. South African medical journal 2013;103(6):402-405

**Study design**

Active case ascertainment

**Country study conducted in**

South Africa

**Was there cocreation of the design of the research study by people with lived experience of FASD?**

No co-creation, some stakeholder involvement

**Number of stages in screening design**

2 stages

**Consent process**

- Opt in at stage 1
- Opt out at stage 2

**Pre-screening variables**

- Height
- Weight
- Head circumference

**Study exclusion criteria**

None reported

**Participation rates**

Overall participation: 93.9% (160/171)

**Non-participation data**

Lack of consent, or absence from school for all follow-up visits

**Type of school provision**

Mainstream

**Sample demographic categories collected (including both those who did and did not participate in the study)**

None

**Participant demographic categories collected (including just those who did participate in the study)**

- Child age
- Child sex

**Did the study use a pre-determined definition of an FASD case?**

Yes

**Guideline used for FASD case definition**

Other: Hoyme et al

**Measurements used: Dysmorphology**

Clinical dysmorphology assessment

**Measurements used: Parent based tools (validation status)**

Not reported

**Measurements used: Child based tools (validation status)**

- Griffiths Mental Development Scale

**Measurements used: Genetic analysis**

None

**Measurements used: Teacher based tools (validation status)**

None

**Children identified as cases seen by a clinician?**

Yes

**Prenatal alcohol exposure data measured**

Not reported

**Prenatal drug exposure data measured**

Not reported

**People individual child results shared with**

- Parents/carers
- School

**Estimated FASD rates**

17.5%

95% CI (12.0% - 24.2%)

**Quality Assessment (QUANDAS-2)**

Patient selection

Was a consecutive or random sample of patients enrolled? Yes

Was a case-control design avoided? Yes

Did the study avoid inappropriate exclusions? Yes

Overall - Could the selection of patient have introduced bias? Level of risk: Low

Applicability - Is there concern that the included patients do not match the review question? Level of risk: Low

Index test(s)

Were the index test results interpreted without knowledge of the results of the reference standard? Yes

If a threshold was used, was it pre-specified? Yes

Overall - Could the conduct or interpretation of the index test have introduced bias? Level of risk: Low

Applicability - Is there concern that the index test, its conduct, or interpretation differ from the review question? Level of risk: Low

Reference standard

Is the reference standard likely to correctly classify the target condition? Yes

Were the reference standard results interpreted without knowledge of the results of the index test? Unclear

Overall - Could the reference standard, its conduct, or its interpretation have introduced bias? Level of risk: Low

Applicability - Is there concern that the target condition as defined by the reference standard does not match the review question? Level of risk: Low

Flow and timing

Was there an appropriate interval between index test(s) and reference standard? Yes

Did all patients receive a reference standard? No - Active case ascertainment design

Did patients receive the same reference standard? Yes

Were all patients included in the analysis? Yes

Overall - Could the patient flow have introduced bias? Level of risk: Low

# 23. Petković 2010

Petković, Giorgie; Barisić, Ingeborg. FAS prevalence in a sample of urban schoolchildren in Croatia.

Reproductive toxicology 2010;29(2):237-241

**Study design**

Active case ascertainment

**Country study conducted in**

Croatia

**Was there cocreation of the design of the research study by people with lived experience of FASD?**

No co-creation, some stakeholder involvement

**Number of stages in screening design**

2 stages

**Consent process**

Opt in

**Pre-screening variables**

- Height
- Weight
- Head circumference

**Study exclusion criteria**

None reported

**Participation rates**

Maternal questionnaire (in this study stage one): 63.04%

**Non-participation data**

None reported

**Type of school provision**

Mainstream

**Sample demographic categories collected (including both those who did and did not participate in the study)**

None

**Participant demographic categories collected (including just those who did participate in the study)**

- Maternal age
- Maternal education
- Household income
- Child age
- Child sex

**Did the study use a pre-determined definition of an FASD case?**

Yes

**Guideline used for FASD case definition**

Other: IOM Hoyme et al

**Measurements used: Dysmorphology**

Clinical dysmorphology assessment

**Measurements used: Parent based tools (validation status)**

None

**Measurements used: Child based tools (validation status)**

None

**Measurements used: Genetic analysis**

None

**Measurements used: Teacher based tools (validation status)**

None

**Children identified as cases seen by a clinician?**

Yes

**Prenatal alcohol exposure data measured**

- Maternal Risk Questionnaire

**Prenatal drug exposure data measured**

- Tobacco

**People individual child results shared with**

Not reported

**Estimated FASD rates**

4.1% (combing FAS and pFAS)

**Quality Assessment (QUANDAS-2)**

Patient selection

Was a consecutive or random sample of patients enrolled? Yes

Was a case-control design avoided? Yes

Did the study avoid inappropriate exclusions? Yes

Overall - Could the selection of patient have introduced bias? Level of risk: Low

Applicability - Is there concern that the included patients do not match the review question? Level of risk: Low

Index test(s)

Were the index test results interpreted without knowledge of the results of the reference standard? Yes

If a threshold was used, was it pre-specified? Yes

Overall - Could the conduct or interpretation of the index test have introduced bias? Level of risk: Low

Applicability - Is there concern that the index test, its conduct, or interpretation differ from the review question? Level of risk: Low

Reference standard

Is the reference standard likely to correctly classify the target condition? Unclear

Were the reference standard results interpreted without knowledge of the results of the index test? Yes

Overall - Could the reference standard, its conduct, or its interpretation have introduced bias? Level of risk: Low

Applicability - Is there concern that the target condition as defined by the reference standard does not match the review question? Level of risk: Unclear

Flow and timing

Was there an appropriate interval between index test(s) and reference standard? Yes

Did all patients receive a reference standard? No – ACA design

Did patients receive the same reference standard? Yes

Were all patients included in the analysis? Yes

Overall - Could the patient flow have introduced bias? Level of risk: Low

# 24. Petković 2013

Petković, Giorgie; Barišić, Ingeborg. Prevalence of fetal alcohol syndrome and maternal characteristics in a sample of schoolchildren from a rural province of Croatia. International journal of environmental research and public health 2013;10(4):1547-1561

**Study design**

Active case ascertainment

**Country study conducted in**

Croatia

**Was there cocreation of the design of the research study by people with lived experience of FASD?**

No co-creation, some stakeholder involvement

**Number of stages in screening design**

2 stages

**Consent process**

Opt in

**Pre-screening variables**

- Height
- Weight
- Head circumference

**Study exclusion criteria**

None reported

**Participation rates**

Maternal questionnaire (for this study stage one): 82.6%

Stage 2: 74.2%

**Non-participation data**

Lack of consent, or unavailable for clinical examination

**Type of school provision**

Mainstream

**Sample demographic categories collected (including both those who did and did not participate in the study)**

None

**Participant demographic categories collected (including just those who did participate in the study)**

- Maternal age
- Maternal education
- Household income
- Child age
- Child sex

**Did the study use a pre-determined definition of an FASD case?**

Yes

**Guideline used for FASD case definition**

Other: Hoyme et al

**Measurements used: Dysmorphology**

Clinical dysmorphology assessment

**Measurements used: Parent based tools (validation status)**

None

**Measurements used: Child based tools (validation status)**

None

**Measurements used: Genetic analysis**

None

**Measurements used: Teacher based tools (validation status)**

None

**Children identified as cases seen by a clinician?**

Yes

**Prenatal alcohol exposure data measured**

- Maternal Risk Questionnaire

**Prenatal drug exposure data measured**

- Tobacco

**People individual child results shared with**

Not reported

**Estimated FASD rates**

Probable: 6.7%

**Quality Assessment (QUANDAS-2)**

Patient selection

Was a consecutive or random sample of patients enrolled? Yes

Was a case-control design avoided? Yes

Did the study avoid inappropriate exclusions? Yes

Overall - Could the selection of patient have introduced bias? Level of risk: Low

Applicability - Is there concern that the included patients do not match the review question? Level of risk: Low

Index test(s)

Were the index test results interpreted without knowledge of the results of the reference standard? Yes

If a threshold was used, was it pre-specified? Yes

Overall - Could the conduct or interpretation of the index test have introduced bias? Level of risk: Low

Applicability - Is there concern that the index test, its conduct, or interpretation differ from the review question? Level of risk: Low

Reference standard

Is the reference standard likely to correctly classify the target condition? Yes

Were the reference standard results interpreted without knowledge of the results of the index test? Yes

Overall - Could the reference standard, its conduct, or its interpretation have introduced bias? Level of risk: Low

Applicability - Is there concern that the target condition as defined by the reference standard does not match the review question? Level of risk: Unclear

Flow and timing

Was there an appropriate interval between index test(s) and reference standard? Yes

Did all patients receive a reference standard? No - ACA design

Did patients receive the same reference standard? No - ACA design

Were all patients included in the analysis? Yes

Overall - Could the patient flow have introduced bias? Level of risk: Low

# 25. Poitra 2003

Poitra, Betty A; Marion, Shirley; Dionne, Marilyn; Wilkie, Esther; Dauphinais, Paul; Wilkie-Pepion, Marma; Martsolf, John T; Klug, Marilyn G; Burd, Larry. A school-based screening program for fetal alcohol syndrome. Neurotoxicology and teratology 2003;25(6):725-729

**Study design**

Active case ascertainment

**Country study conducted in**

Croatia

**Was there cocreation of the design of the research study by people with lived experience of FASD?**

Not reported

**Number of stages in screening design**

2 stages

**Consent process**

Opt out

**Pre-screening variables**

None used

**Study exclusion criteria**

None reported

**Participation rates**

Screened at stage 1: 98% (1384 children screened overall)

**Non-participation data**

Reasons for non-participation were not reported

**Type of school provision**

Mainstream

**Sample demographic categories collected (including both those who did and did not participate in the study)**

None

**Participant demographic categories collected (including just those who did participate in the study)**

None

**Did the study use a pre-determined definition of an FASD case?**

Yes

**Guideline used for FASD case definition**

Other: The diagnostic criteria utilized were from Sokol and Clarren. Study also
retrospectively applied the criteria from the Institute of Medicine Report on Fetal Alcohol Syndrome

**Measurements used: Dysmorphology**

Clinical dysmorphology assessment

**Measurements used: Parent based tools (validation status)**

None

**Measurements used: Child based tools (validation status)**

- FAS Screen - a 32-item screening test

**Measurements used: Genetic analysis**

None

**Measurements used: Teacher based tools (validation status)**

None

**Children identified as cases seen by a clinician?**

Yes

**Prenatal alcohol exposure data measured**

- Self-reported maternal history

**Prenatal drug exposure data measured**

Not reported

**People individual child results shared with**

- GP
- School

**Estimated FASD rates**

0.50% 95% CI (0.2%, 1.0%)

**Quality Assessment (QUANDAS-2)**

Patient selection

Was a consecutive or random sample of patients enrolled? Yes

Was a case-control design avoided? Yes

Did the study avoid inappropriate exclusions? Yes

Overall - Could the selection of patient have introduced bias? Level of risk: Low

Applicability - Is there concern that the included patients do not match the review question? Level of risk: Low

Index test(s)

Were the index test results interpreted without knowledge of the results of the reference standard? Yes

If a threshold was used, was it pre-specified? Unclear - Low level of detail reported about interpretations of the index test

Overall - Could the conduct or interpretation of the index test have introduced bias? Level of risk: Unclear - Low level of detail reported about interpretations of the index test

Applicability - Is there concern that the index test, its conduct, or interpretation differ from the review question? Level of risk: Low

Reference standard

Is the reference standard likely to correctly classify the target condition? Unclear - Only FAS diagnoses were included, not those for all FASD

Were the reference standard results interpreted without knowledge of the results of the index test? Unclear - Not reported in the study

Overall - Could the reference standard, its conduct, or its interpretation have introduced bias? Level of risk: Unclear - Only FAS diagnoses were included, not those for all FASD

Applicability - Is there concern that the target condition as defined by the reference standard does not match the review question? Level of risk: Unclear – Only FAS diagnoses were included, not those for all FASD

Flow and timing

Was there an appropriate interval between index test(s) and reference standard? Yes

Did all patients receive a reference standard? No - Only those with suspected FAS

Did patients receive the same reference standard? Yes

Were all patients included in the analysis? Yes

Overall - Could the patient flow have introduced bias? Level of risk: High - No account was taken of the risk of undiagnosed FAS in those not referred for the reference standard

# 26. Popova 2019

Popova, Svetlana; Lange, Shannon; Poznyak, Vladimir; Chudley, Albert E; Shield, Kevin D; Reynolds, James N; Murray, Margaret; Rehm, Jürgen. Population-based prevalence of fetal alcohol spectrum disorder in Canada. BMC public health 2019;19(1):845

**Study design**

Active case ascertainment

**Country study conducted in**

Canada

**Was there cocreation of the design of the research study by people with lived experience of FASD?**

No co-creation, some stakeholder involvement

**Number of stages in screening design**

2 stages

**Consent process**

Opt in

**Pre-screening variables**

- Height
- Weight
- Head circumference

**Study exclusion criteria**

None reported

**Participation rates**

First stage: 2555/8209 (31.0%)

Second stage: 762/793 (96.0%)

**Non-participation data**

First stage: Lack of consent, absent from school on day of assessments

Second dates: Lost to follow-up, withdrew from study

**Type of school provision**

Mainstream

**Sample demographic categories collected (including both those who did and did not participate in the study)**

None

**Participant demographic categories collected (including just those who did participate in the study)**

- Maternal age
- Maternal education
- Maternal ethnicity
- Household income
- Child age
- Child sex

**Did the study use a pre-determined definition of an FASD case?**

Yes

**Guideline used for FASD case definition**

Canadian

**Measurements used: Dysmorphology**

Clinical dysmorphology assessment

**Measurements used: Parent based tools (validation status)**

- Child Behaviour Checklist

**Measurements used: Child based tools (validation status)**

- WISC V (validated)
- NEPSY (validated)
- WASI II

**Measurements used: Genetic analysis**

None

**Measurements used: Teacher based tools (validation status)**

None

**Children identified as cases seen by a clinician?**

Not reported

**Prenatal alcohol exposure data measured**

- Self-reported maternal history

**Prenatal drug exposure data measured**

- Illicit drugs
- Tobacco

**People individual child results shared with**

Not reported

**Estimated FASD rates**

Probable FASD: 2.93% (95% CI 12.4%-56.2%)

**Quality Assessment (QUANDAS-2)**

Patient selection

Was a consecutive or random sample of patients enrolled? Yes

Was a case-control design avoided? Yes

Did the study avoid inappropriate exclusions? Yes

Overall - Could the selection of patient have introduced bias? Level of risk: Low

Applicability - Is there concern that the included patients do not match the review question? Level of risk: Low

Index test(s)

Were the index test results interpreted without knowledge of the results of the reference standard? Unclear

If a threshold was used, was it pre-specified? Yes

Overall - Could the conduct or interpretation of the index test have introduced bias? Level of risk: Low

Applicability - Is there concern that the index test, its conduct, or interpretation differ from the review question? Level of risk: Low

Reference standard

Is the reference standard likely to correctly classify the target condition? Yes

Were the reference standard results interpreted without knowledge of the results of the index test? Yes

Overall - Could the reference standard, its conduct, or its interpretation have introduced bias? Level of risk: Low

Applicability - Is there concern that the target condition as defined by the reference standard does not match the review question? Level of risk: Low

Flow and timing

Was there an appropriate interval between index test(s) and reference standard? Yes

Did all patients receive a reference standard? No - Only those suspected at earlier stages, plus a random sample of other children

Did patients receive the same reference standard? Yes

Were all patients included in the analysis? Yes

Overall - Could the patient flow have introduced bias? Level of risk: Low - Although not all children received a reference standard, giving one to a random subsample mitigates any risks of bias

# 27. Urban 2008

Urban, Michael; Chersich, Matthew F; Fourie, Leigh-Anne; Chetty, Candice; Olivier, Leana; Viljoen, Denis. Fetal alcohol syndrome among grade 1 schoolchildren in Northern Cape Province: prevalence and risk factors. South African medical journal. 2008;98(11):877-882

**Study design**

Active case ascertainment

**Country study conducted in**

South Africa

**Was there cocreation of the design of the research study by people with lived experience of FASD?**

None reported

**Number of stages in screening design**

2 stages

**Consent process**

- Opt in at stage 1
- Opt out at stage 2

**Pre-screening variables**

- Height
- Weight
- Head circumference

**Study exclusion criteria**

None

**Participation rates**

Not reported

**Non-participation data**

Not reported

**Type of school provision**

Mainstream

**Sample demographic categories collected (including both those who did and did not participate in the study)**

- Child age
- Child sex
- Household income
- Deprivation level of school area

**Participant demographic categories collected (including just those who did participate in the study)**

- Maternal age
- Maternal education
- Household income

**Did the study use a pre-determined definition of an FASD case?**

Yes

**Guideline used for FASD case definition**

Other: Institute of Medicine (IOM) criteria Hoyme et al

**Measurements used: Dysmorphology**

Clinical dysmorphology assessment

**Measurements used: Parent based tools (validation status)**

None

**Measurements used: Child based tools (validation status)**

- WISC V (validated)
- NEPSY (validated)
- Tests of Reception Grammar
- Ravens Coloured Progressive Matrices
- DAP

**Measurements used: Genetic analysis**

None

**Measurements used: Teacher based tools (validation status)**

Not reported

**Children identified as cases seen by a clinician?**

Not reported

**Prenatal alcohol exposure data measured**

- Maternal Risk Questionnaire

**Prenatal drug exposure data measured**

Not reported

**People individual child results shared with**

- Parents/carers
- GP
- School

**Estimated FASD rates**

Area 1: 11.9% (95% CI 9.32%-14.99%)
Area 2: 7.4% (95% CI 6.10%-9.03%)

**Quality Assessment (QUANDAS-2)**

Patient selection

Was a consecutive or random sample of patients enrolled? Yes

Was a case-control design avoided? Yes

Did the study avoid inappropriate exclusions? Yes

Overall - Could the selection of patient have introduced bias? Level of risk: Low

Applicability - Is there concern that the included patients do not match the review question? Level of risk: Low

Index test(s)

Were the index test results interpreted without knowledge of the results of the reference standard? Yes

If a threshold was used, was it pre-specified? Yes

Overall - Could the conduct or interpretation of the index test have introduced bias? Level of risk: Low

Applicability - Is there concern that the index test, its conduct, or interpretation differ from the review question? Level of risk: Unclear

Reference standard

Is the reference standard likely to correctly classify the target condition? Yes

Were the reference standard results interpreted without knowledge of the results of the index test? Yes

Overall - Could the reference standard, its conduct, or its interpretation have introduced bias? Level of risk: Low

Applicability - Is there concern that the target condition as defined by the reference standard does not match the review question? Level of risk: Unclear

Flow and timing

Was there an appropriate interval between index test(s) and reference standard? Yes

Did all patients receive a reference standard? No - ACA design

Did patients receive the same reference standard? Yes

Were all patients included in the analysis? Yes

Overall - Could the patient flow have introduced bias? Level of risk: Low

# 28. Urban 2015

CUrban MF, Olivier L, Viljoen D, Lombard C, Louw JG, Drotsky LM, Temmerman M, Chersich MF. Prevalence of fetal alcohol syndrome in a South African city with a predominantly Black African population. Alcohol Clin Exp Res. 2015 Jun;39(6):1016-26. doi: 10.1111/acer.12726. Epub 2015 May 2. PMID: 25941030.

**Study design**

Active case ascertainment

**Country study conducted in**

South Africa

**Was there cocreation of the design of the research study by people with lived experience of FASD?**

None reported

**Number of stages in screening design**

3 stages

**Consent process**

Opt out

**Pre-screening variables**

- Height
- Weight
- Head circumference

**Study exclusion criteria**

None reported

**Participation rates**

Stage 1: 95.1% (1510/1587)

Stage 2: 99.0% (487/492)

Stage 3: 98.3% (113/115)

**Non-participation data**

Primary reason was lack of consent

**Type of school provision**

Mainstream

**Sample demographic categories collected (including both those who did and did not participate in the study)**

- Maternal ethnicity

**Participant demographic categories collected (including just those who did participate in the study)**

- Maternal age
- Maternal ethnicity
- Maternal education
- Household income

**Did the study use a pre-determined definition of an FASD case?**

Yes

**Guideline used for FASD case definition**

Hoyme et al 2005

**Measurements used: Dysmorphology**

Clinical dysmorphology assessment

**Measurements used: Parent based tools (validation status)**

None

**Measurements used: Child based tools (validation status)**

- Griffiths Mental Development Scale

**Measurements used: Genetic analysis**

None

**Measurements used: Teacher based tools (validation status)**

None

**Children identified as cases seen by a clinician?**

No

**Prenatal alcohol exposure data measured**

- Maternal Risk Questionnaire

**Prenatal drug exposure data measured**

- Tobacco use during pregnancy

**People individual child results shared with**

Not reported

**Estimated FASD rates**

6.4%

**Quality Assessment (QUANDAS-2)**

Patient selection

Was a consecutive or random sample of patients enrolled? Yes

Was a case-control design avoided? Yes

Did the study avoid inappropriate exclusions? Yes

Overall - Could the selection of patient have introduced bias? Level of risk: Low

Applicability - Is there concern that the included patients do not match the review question? Level of risk: Low

Index test(s)

Were the index test results interpreted without knowledge of the results of the reference standard? Yes

If a threshold was used, was it pre-specified? Yes

Overall - Could the conduct or interpretation of the index test have introduced bias? Level of risk: Low

Applicability - Is there concern that the index test, its conduct, or interpretation differ from the review question? Level of risk: Low

Reference standard

Is the reference standard likely to correctly classify the target condition? Unclear - No agreed diagnostic criteria for FASD

Were the reference standard results interpreted without knowledge of the results of the index test? No - Index tests form part of the reference standard

Overall - Could the reference standard, its conduct, or its interpretation have introduced bias? Level of risk: Unclear - No agreed diagnostic criteria for FASD

Applicability - Is there concern that the target condition as defined by the reference standard does not match the review question? Level of risk: Unclear - No agreed diagnostic criteria for FASD

Flow and timing

Was there an appropriate interval between index test(s) and reference standard? Yes

Did all patients receive a reference standard? Yes

Did patients receive the same reference standard? Yes

Were all patients included in the analysis? No - All participants not consenting to be in the study were assumed not to have FASD

Overall - Could the patient flow have introduced bias? Level of risk: High - All participants not consenting to be in the study were assumed not to have FASD, which is likely to bias prevalence estimates

# 29. Viljoen 2005

Viljoen, Denis L; Gossage, J Phillip; Brooke, Lesley; Adnams, Colleen M; Jones, Kenneth L; Robinson, Luther K; Hoyme, H Eugene; Snell, Cudore; Khaole, Nathaniel C O; Kodituwakku, Piyadasa; Asante, Kwadwo Ohene; Findlay, Richard; Quinton, Barbara; Marais, Anna-Susan; Kalberg, Wendy O; May, Philip A. Fetal alcohol syndrome epidemiology in a South African community: a second study of a very high prevalence area. Journal of studies on alcohol 2005;66(5):593-604

**Study design**

Active case ascertainment

**Country study conducted in**

South Africa

**Was there cocreation of the design of the research study by people with lived experience of FASD?**

Not reported

**Number of stages in screening design**

2 stages

**Consent process**

- Opt in at stage 1
- Opt out in at stage 2

**Pre-screening variables**

- Height
- Weight
- Head circumference

**Study exclusion criteria**

None reported

**Participation rates**

Stage 1: 93.6% (863/922)

Stage 2: 88.2% (210/238)

**Non-participation data**

Stage 1: Lack of consent

Stage 2: Not available for interview

**Type of school provision**

Mainstream

**Sample demographic categories collected (including both those who did and did not participate in the study)**

None

**Participant demographic categories collected (including just those who did participate in the study)**

- Maternal ethnicity

**Did the study use a pre-determined definition of an FASD case?**

Yes

**Guideline used for FASD case definition**

Other: Hoyme

**Measurements used: Dysmorphology**

Clinical dysmorphology assessment

**Measurements used: Parent based tools (validation status)**

- Personal Behaviours Checklist

**Measurements used: Child based tools (validation status)**

- Tests of Reception Grammar
- Ravens Coloured Progressive Matrices

**Measurements used: Genetic analysis**

Not reported

**Measurements used: Teacher based tools (validation status)**

None

**Children identified as cases seen by a clinician?**

No

**Prenatal alcohol exposure data measured**

- Maternal Risk Questionnaire

**Prenatal drug exposure data measured**

- Tobacco

**People individual child results shared with**

Not reported

**Estimated FASD rates**

FAS: 6.52%-7.42%

**Quality Assessment (QUANDAS-2)**

Patient selection

Was a consecutive or random sample of patients enrolled? Yes

Was a case-control design avoided? Yes

Did the study avoid inappropriate exclusions? Yes

Overall - Could the selection of patient have introduced bias? Level of risk: Low

Applicability - Is there concern that the included patients do not match the review question? Level of risk: Low

Index test(s)

Were the index test results interpreted without knowledge of the results of the reference standard? Yes

If a threshold was used, was it pre-specified? Yes

Overall - Could the conduct or interpretation of the index test have introduced bias? Level of risk: Low

Applicability - Is there concern that the index test, its conduct, or interpretation differ from the review question? Level of risk: Low

Reference standard

Is the reference standard likely to correctly classify the target condition? Yes

Were the reference standard results interpreted without knowledge of the results of the index test? Yes

Overall - Could the reference standard, its conduct, or its interpretation have introduced bias? Level of risk: Low

Applicability - Is there concern that the target condition as defined by the reference standard does not match the review question? Level of risk: High - Full FAS only, not FASD

Flow and timing

Was there an appropriate interval between index test(s) and reference standard? Yes

Did all patients receive a reference standard? No - Design prevented all patients being assessed

Did patients receive the same reference standard? Yes

Were all patients included in the analysis? Yes

Overall - Could the patient flow have introduced bias? Level of risk: Low
