# Supplementary material for: Fetal Alcohol Spectrum Disorder Prevalence Studies in School Settings: A Global Systematic Review of Methods, Measures, Strengths and Limitations
Source: Drug Alcohol Rev. 2026 Sep 10;45(6):e70233. doi: 10.1111/dar.70233 (PMC13559213; doi:10.1111/dar.70233)
Supplement: Supplementary file 2 [file DAR-45-0-s002.docx]

**MEDLINE**

(MW "fetal alcohol spectrum disorders" OR TI fetal N3 alcohol OR AB fetal N3 alcohol OR TI fetal N3 ethanol OR AB fetal N3 ethanol OR TI "in utero" N3 alcohol OR AB "in utero" N3 alcohol OR TI "in utero" N3 ethanol OR AB "in utero" N3 ethanol OR TI pregn* N3 alcohol OR AB pregn* N3 alcohol OR TI pregn* N3 ethanol OR AB pregn* N3 ethanol OR TI pregn* N3 drink* OR AB pregn* N3 drink* OR TI "prenatal alcohol exposure" OR AB "prenatal alcohol exposure" OR TI FASD OR AB FASD OR TI "fetal alcohol spectrum disorder*" OR AB "fetal alcohol spectrum disorder*" OR TI "fetal alcohol syndrome" OR AB "fetal alcohol syndrome*" OR TI ARND OR AB ARND OR TI "alcohol related neurodevelopmental disorder" OR AB "alcohol related neurodevelopmental disorder" OR TI microceph* N3 alcohol OR AB microceph* N3 alcohol OR TI microceph* N3 ethanol OR AB microceph* N3 ethanol OR TI neonatal N3 alcohol OR AB neonatal N3 alcohol OR TI neonatal N3 ethanol OR AB neonatal N3 ethanol OR TI "neurodevelopmental disorder" N3 alcohol OR AB "neurodevelopmental disorder" N3 alcohol)

AND

(TI ( "elementary education" OR "elementary school*" OR "elementary student*" OR "elementary grad*" OR "primary education" OR "primary school*" OR "primary student*" OR "primary grad*" OR "secondary education" OR "secondary school*" OR "secondary student*" OR "secondary grad*" OR "middle school*" OR "middle-school*" OR "high school*" OR "high-school*" OR student* OR school* OR education ) OR AB ( "elementary education" OR "elementary school*" OR "elementary student*" OR "elementary grad*" OR "primary education" OR "primary school*" OR "primary student*" OR "primary grad*" OR "secondary education" OR "secondary school*" OR "secondary student*" OR "secondary grad*" OR "middle school*" OR "middle-school*" OR "high school*" OR "high-school*" OR student* OR school* OR education ))

AND

(TI ( instrument* OR measur* OR scale* OR questionnaire* OR survey* OR assess* OR screen* OR audit OR prevalence OR sensitivity OR specificity OR predictive value ) OR AB ( instrument* OR measur* OR scale* OR questionnaire* OR survey* OR assess* OR screen* OR audit OR prevalence OR sensitivity OR specificity OR predictive value))

**EBSCOhost CINAHL**

(MM "Fetal Alcohol Spectrum Disorder") OR (MM "Fetal Alcohol Spectrum Disorders") OR "Prenatal Exposure Delayed Effects" OR (MM "FASD") OR "prenatal exposure delayed effects" OR "prenatal alcohol" OR "prenatal ethanol" OR (in utero ethanol) OR (pregn* alcohol) OR (pregn* ethanol) OR (pregn* drinking) OR (MM "prenatal alcohol exposure") OR "Prenatal Exposure Delayed Effects" OR (MM "Fetal Alcohol Syndrome") OR "alcohol related neurodevelopmental disorder" OR ("alcohol related neurological difficulties") OR (“fetal alcohol") OR (“microceph*,(alcohol or ethanol)) OR ((fetal or neonat* or prenat*) (alcohol or ethanol)) OR (“neurodevelopmental disorder” (alcohol or ethanol)) OR (MM ”FAS") OR (MM "pFAS") OR (MM "ARND") OR (MM "FASD")

AND

"elementary education" OR "elementary school*" OR "elementary student*" OR "elementary grad*" OR "primary education" OR "primary school*" OR "primary student*" OR "primary grad*" OR "secondary education" OR "secondary school*" OR "secondary student*" OR "secondary grad*" OR "middle school*" OR "middle-school*" OR "high school*" OR "high-school*" OR student* OR school* OR education

AND

instrument* OR measur* OR scale* OR questionnaire* OR survey* OR assess* OR screen* OR audit OR prevalence OR sensitivity OR specificity OR predictive value

**PyscINFO**

(TI fetal N3 alcohol OR AB fetal N3 alcohol OR TI fetal N3 ethanol OR AB fetal N3 ethanol OR TI "in utero" N3 alcohol OR AB "in utero" N3 alcohol OR TI "in utero" N3 ethanol OR AB "in utero" N3 ethanol OR TI pregn* N3 alcohol OR AB pregn* N3 alcohol OR TI pregn* N3 ethanol OR AB pregn* N3 ethanol OR TI pregn* N3 drink* OR AB pregn* N3 drink* OR TI "prenatal alcohol exposure" OR AB "prenatal alcohol exposure" OR TI FASD OR AB FASD OR TI "fetal alcohol spectrum disorder*" OR AB "fetal alcohol spectrum disorder*" OR TI "fetal alcohol syndrome" OR AB "fetal alcohol syndrome*" OR TI ARND OR AB ARND OR TI "alcohol related neurodevelopmental disorder" OR AB "alcohol related neurodevelopmental disorder" OR TI microceph* N3 alcohol OR AB microceph* N3 alcohol OR TI microceph* N3 ethanol OR AB microceph* N3 ethanol OR TI neonatal N3 alcohol OR AB neonatal N3 alcohol OR TI neonatal N3 ethanol OR AB neonatal N3 ethanol OR TI "neurodevelopmental disorder" N3 alcohol OR AB "neurodevelopmental disorder" N3 alcohol)

AND

(TI ( "elementary education" OR "elementary school*" OR "elementary student*" OR "elementary grad*" OR "primary education" OR "primary school*" OR "primary student*" OR "primary grad*" OR "secondary education" OR "secondary school*" OR "secondary student*" OR "secondary grad*" OR "middle school*" OR "middle-school*" OR "high school*" OR "high-school*" OR student* OR school* OR education ) OR AB ( "elementary education" OR "elementary school*" OR "elementary student*" OR "elementary grad*" OR "primary education" OR "primary school*" OR "primary student*" OR "primary grad*" OR "secondary education" OR "secondary school*" OR "secondary student*" OR "secondary grad*" OR "middle school*" OR "middle-school*" OR "high school*" OR "high-school*" OR student* OR school* OR education))

AND

(TI ( instrument* OR measur* OR scale* OR questionnaire* OR survey* OR assess* OR screen* OR audit OR prevalence OR sensitivity OR specificity OR predictive value ) OR AB ( instrument* OR measur* OR scale* OR questionnaire* OR survey* OR assess* OR screen* OR audit OR prevalence OR sensitivity OR specificity OR predictive value))

**Child Development & Adolescent Studies**

(TI fetal N3 alcohol OR AB fetal N3 alcohol OR TI fetal N3 ethanol OR AB fetal N3 ethanol OR TI "in utero" N3 alcohol OR AB "in utero" N3 alcohol OR TI "in utero" N3 ethanol OR AB "in utero" N3 ethanol OR TI pregn* N3 alcohol OR AB pregn* N3 alcohol OR TI pregn* N3 ethanol OR AB pregn* N3 ethanol OR TI pregn* N3 drink* OR AB pregn* N3 drink* OR TI "prenatal alcohol exposure" OR AB "prenatal alcohol exposure" OR TI FASD OR AB FASD OR TI "fetal alcohol spectrum disorder*" OR AB "fetal alcohol spectrum disorder*" OR TI "fetal alcohol syndrome" OR AB "fetal alcohol syndrome*" OR TI ARND OR AB ARND OR TI "alcohol related neurodevelopmental disorder" OR AB "alcohol related neurodevelopmental disorder" OR TI microceph* N3 alcohol OR AB microceph* N3 alcohol OR TI microceph* N3 ethanol OR AB microceph* N3 ethanol OR TI neonatal N3 alcohol OR AB neonatal N3 alcohol OR TI neonatal N3 ethanol OR AB neonatal N3 ethanol OR TI "neurodevelopmental disorder" N3 alcohol OR AB "neurodevelopmental disorder" N3 alcohol)

AND

(TI ( "elementary education" OR "elementary school*" OR "elementary student*" OR "elementary grad*" OR "primary education" OR "primary school*" OR "primary student*" OR "primary grad*" OR "secondary education" OR "secondary school*" OR "secondary student*" OR "secondary grad*" OR "middle school*" OR "middle-school*" OR "high school*" OR "high-school*" OR student* OR school* OR education ) OR AB ( "elementary education" OR "elementary school*" OR "elementary student*" OR "elementary grad*" OR "primary education" OR "primary school*" OR "primary student*" OR "primary grad*" OR "secondary education" OR "secondary school*" OR "secondary student*" OR "secondary grad*" OR "middle school*" OR "middle-school*" OR "high school*" OR "high-school*" OR student* OR school* OR education))

AND

(TI ( instrument* OR measur* OR scale* OR questionnaire* OR survey* OR assess* OR screen* OR audit OR prevalence OR sensitivity OR specificity OR predictive value ) OR AB ( instrument* OR measur* OR scale* OR questionnaire* OR survey* OR assess* OR screen* OR audit OR prevalence OR sensitivity OR specificity OR predictive value))

Cochrane

"fetal alcohol spectrum disorder*" OR FASD OR "fetal alcohol syndrome*" OR "alcohol related neurodevelopmental disorder*" OR ARND OR "prenatal alcohol exposure" OR (fetal NEAR/3 alcohol) OR (fetal NEAR/3 ethanol) OR ("in utero" NEAR/3 alcohol) OR ("in utero" NEAR/3 ethanol) OR (pregn* NEAR/3 alcohol) OR (pregn* NEAR/3 ethanol) OR (pregn* NEAR/3 drink*) OR (microceph* NEAR/3 alcohol) OR (microceph* NEAR/3 ethanol) OR (neonatal NEAR/3 alcohol) OR (neonatal NEAR/3 ethanol) OR ("neurodevelopmental disorder*" NEAR/3 alcohol)

AND

"elementary education" OR "elementary school*" OR "elementary student*" OR "elementary grad*" OR "primary education" OR "primary school*" OR "primary student*" OR "primary grad*" OR "secondary education" OR "secondary school*" OR "secondary student*" OR "secondary grad*" OR "middle school*" OR "middle-school*" OR "high school*" OR "high-school*" OR student* OR school* OR education

AND

instrument* OR measur* OR scale* OR questionnaire* OR survey* OR assess* OR screen* OR audit OR prevalence OR sensitivity OR specificity OR "predictive value*"
