# Supplementary material for: Fetal Alcohol Spectrum Disorder Prevalence Studies in School Settings: A Global Systematic Review of Methods, Measures, Strengths and Limitations
Source: Drug Alcohol Rev. 2026 Sep 10;45(6):e70233. doi: 10.1111/dar.70233 (PMC13559213; doi:10.1111/dar.70233)
Supplement: Supplementary file 3 [file DAR-45-0-s003.docx]

**Supplementary appendix 1 – Studies excluded at full-text review**

| **Authors** | **Title** | **Reason for exclusion** |
| --- | --- | --- |
| Flannigan, Katherine; Poole, Nancy; Cook, Jocelynn; Unsworth, Kathy | Sex-related differences among individuals assessed for fetal alcohol spectrum disorder in Canada. | Study does not contain a population of people being screened for FASD in a school setting. |
| Montag, Annika C; Jones, Kenneth Lyons; Del Campo, Miguel; Akshoomoff, Natacha; Coles, Claire D; Kable, Julie A; Hernandez, John-Gregorey; Chambers, Christina D | Fetal alcohol spectrum disorders and access to regional center services in San Diego County. | Study does not contain a population of people being screened for FASD in a school setting. |
| Muggli, Evelyne; Halliday, Jane; Elliott, Elizabeth J; Penington, Anthony; Thompson, Deanne; Spittle, Alicia Jane; Forster, Della; Lewis, Sharon; Hearps, Stephen; Anderson, Peter J | Cohort profile: early school years follow-up of the Asking Questions about Alcohol in Pregnancy Longitudinal Study in Melbourne, Australia (AQUA at 6). | Study does not contain a population of people being screened for FASD in a school setting. |
| Wynn, Adriane; Rotheram-Borus, Mary Jane; Davis, Emily; le Roux, Ingrid; Almirol, Ellen; O'Connor, Mary; Tomlinson, Mark | Identifying fetal alcohol spectrum disorder among South African children at aged 1 and 5 years. | Study does not contain a population of people being screened for FASD in a school setting. |
| Taylor, Nicole M; Enns, Leah N | Age-related differences in neuropsychological assessment of fetal alcohol spectrum disorder: a cross-sectional study. | Study does not contain a population of people being screened for FASD in a school setting. |
| Wagner, Bree; Fitzpatrick, James P; Mazzucchelli, Trevor G; Symons, Martyn; Carmichael Olson, Heather; Jirikowic, Tracy; Cross, Donna; Wright, Edie; Adams, Emma; Carter, Maureen; Bruce, Kaashifah; Latimer, Jane | Study protocol for a self-controlled cluster randomised trial of the Alert Program to improve self-regulation and executive function in Australian Aboriginal children with fetal alcohol spectrum disorder. | Study protocol without any collected data. |
| Jacobson, Sandra W; Stanton, Mark E; Dodge, Neil C; Pienaar, Mariska; Fuller, Douglas S; Molteno, Christopher D; Meintjes, Ernesta M; Hoyme, H Eugene; Robinson, Luther K; Khaole, Nathaniel; Jacobson, Joseph L | Impaired delay and trace eyeblink conditioning in school-age children with fetal alcohol syndrome. | Study does not contain a population of people being screened for FASD in a school setting. |
| May, Philip A; Gossage, J Phillip; Marais, Anna-Susan; Hendricks, Loretta S; Snell, Cudore L; Tabachnick, Barbara G; Stellavato, Chandra; Buckley, David G; Brooke, Lesley E; Viljoen, Denis L | Maternal risk factors for fetal alcohol syndrome and partial fetal alcohol syndrome in South Africa: a third study. | Secondary analysis of data already included within another paper in this review. |
| AragÃ³n, Alfredo S; Coriale, Giovanna; Fiorentino, Daniela; Kalberg, Wendy O; Buckley, David; Gossage, J Phillip; Ceccanti, Mauro; Mitchell, Elisha R; May, Philip A | Neuropsychological characteristics of Italian children with fetal alcohol spectrum disorders. | Study does not report any data on screening for FASD. |
| Goldschmidt, L; Richardson, G A; Stoffer, D S; Geva, D; Day, N L | Prenatal alcohol exposure and academic achievement at age six: a nonlinear fit. | Study does not contain a population of people being screened for FASD in a school setting. |
| Mena, M; Nazal, R; Albornoz, C; Pettinelli, H; VelÃ¡squez, P; Soza, G | Fetal alcohol syndrome: prevalence in 4 special education schools in Chile. | Study not available in English. |
| National Institute on Alcohol Abuse and Alcoholism | State watch. School district surveys first grade students for FASD. | Not a peer-reviewed publication. |
| Duncan, Charles Randy | The development and validation of the screening Screening Test for the Early Prediction of School Success (STEPSS): A screen of cognitive functioning in four- and five-year old children with varying health conditions. | Study does not report any data on screening for FASD. |
| Chałupka, Anna; Sobieska, Magdalena | Review of literature and diagnostic tools for assessing psychomotor development in infants exposed to fetal alcohol exposure. | Study does not report any data on screening for FASD |
